# Developmental Designs and Adult Functions of Cortical Maps in Multiple Modalities: Perception, Attention, Navigation, Numbers, Streaming, Speech, and Cognition

**DOI:** 10.3389/fninf.2020.00004

**Published:** 2020-02-06

**Authors:** Stephen Grossberg

**Affiliations:** Center for Adaptive Systems, Graduate Program in Cognitive and Neural Systems, Departments of Mathematics & Statistics, Psychological & Brain Sciences, and Biomedical Engineering, Boston University, Boston, MA, United States

**Keywords:** adaptive resonance theory, ocular dominance column, auditory stream, place-value number, speaker normalization, reinforcement learning, navigation, working memory

## Abstract

This article unifies neural modeling results that illustrate several basic design principles and mechanisms that are used by advanced brains to develop cortical maps with multiple psychological functions. One principle concerns how brains use a *strip map* that simultaneously enables one feature to be represented throughout its extent, as well as an ordered array of another feature at different positions of the strip. Strip maps include circuits to represent ocular dominance and orientation columns, place-value numbers, auditory streams, speaker-normalized speech, and cognitive working memories that can code repeated items. A second principle concerns how feature detectors for multiple functions develop in topographic maps, including maps for optic flow navigation, reinforcement learning, motion perception, and category learning at multiple organizational levels. A third principle concerns how brains exploit a spatial gradient of cells that respond at an ordered sequence of different rates. Such a rate gradient is found along the dorsoventral axis of the entorhinal cortex, whose lateral branch controls the development of time cells, and whose medial branch controls the development of grid cells. Populations of time cells can be used to learn how to adaptively time behaviors for which a time interval of hundreds of milliseconds, or several seconds, must be bridged, as occurs during trace conditioning. Populations of grid cells can be used to learn hippocampal place cells that represent the large spaces in which animals navigate. A fourth principle concerns how and why all neocortical circuits are organized into layers, and how functionally distinct columns develop in these circuits to enable map development. A final principle concerns the role of Adaptive Resonance Theory top-down matching and attentional circuits in the dynamic stabilization of early development and adult learning. Cortical maps are modeled in visual, auditory, temporal, parietal, prefrontal, entorhinal, and hippocampal cortices.

## Cortical Maps: A Basic Principle of Cortical Design

The editors of this *Frontiers* Research Topic, Nick Swindale and Geoffrey Goodhill, have posed several basic questions about cortical maps, notably concerning whether or not, despite being ubiquitous in advanced brains, they have functional significance. In particular, these authors wrote: “while maps seem to be ubiquitous in the primary sensory cortical areas, many questions about their significance remain. Might they simply be an epiphenomenon of development with no real functional significance? How widespread are maps in the cortex? For example are there maps of speech properties in Broca’s area? Are there maps in the frontal cortex?… Orientation columns are also a puzzle, because it seems they can develop in the absence of natural visual stimulation but it is not clear how this could happen. Retinal waves may not have enough structure nor are they easy for models based on them to explain how matched preferences can develop in the two eyes. Their periodic structure has also been especially hard to capture.”

This article proposes answers to all these questions. It does so by unifying a series of modeling studies that were carried out during the past 40 years by the author with multiple colleagues. The article focuses upon these research streams because, to the best of my knowledge, the resultant models, after multiple stages of development and refinement, come closer to principled theories of their large targeted databases than alternative models in the literature. These multiple stages of model evolution have accumulated and satisfied computational and experimental constraints that competing models have not, at least to the present time.

This theoretical approach tries to at least partially alleviate a general problem in modeling brain models of psychological phenomena: models that propose explanations of small neurobiological data sets often cannot survive under the weight of accumulating interdisciplinary constraints. For example, in modeling visual cortical development, several models may simulate small data sets about the simplest properties of simple and complex cells. Some analyses may even cast doubt on the existence of separate classes of simple and complex cells; e.g., Mechler and Ringach (2002). However, they may fail to show how the results of their analyses can support conscious visual perception, which is the evolutionary outcome of vision.

The perspective taken in this article is to be guided by all available evidence to attempt to construct a principled computational theory that is powerful enough to explain psychological and neurobiological data on multiple levels of organization, ranging from single-cell properties to organismic behaviors. Our own models have thus been shaped by the weight of both psychological and neurobiological constraints to provide accumulating evidence for the validity of their main design principles, mechanisms, circuits, and architectures. The article will also describe various alternative models as part of its exposition, and will use this review to compare and contrast them with the models that are its focus.

As noted above, the models that have emerged from this process of conceptual and mechanistic evolution suggest answers to all the questions in the first paragraph, in addition to others about cortical organization in general and cortical maps in particular, including the organization of cortical maps within the characteristic *layers* of all neocortical circuits. Previous articles from the author and his colleagues have shown how variations of the same canonical laminar cortical architecture can be used to explain and simulate neurobiological and psychological data about vision, speech and language, and cognitive information processing. The current article suggests how and why this laminar organization, sometimes called Laminar Computing, constrains how cortical maps form. Few, if any, alternative models of cortical map formation have considered how maps develop within and across this canonical cortical laminar architecture.

Within this unifying framework, the exposition proposes how a small number of design principles and mechanisms have been used in neural models to explain and unify the explanation of psychological and neurobiological data for brain functions as diverse as:

- visual retinogeniculate, thalamocortical, and corticocortical development, perception, attention, and categorization (Grossberg and Levine, 1975; Grossberg, 1975b, 1976a; Grunewald and Grossberg, 1998; Olson and Grossberg, 1998; Grossberg and Kelly, 1999; Grossberg and Raizada, 2000; Kelly and Grossberg, 2000; Grossberg and Williamson, 2001; Raizada and Grossberg, 2001, 2003; Grossberg and Grunewald, 2002; Grossberg and Seitz, 2003; Grossberg and Swaminathan, 2004; Cao and Grossberg, 2005, 2012; Markowitz et al., 2012);
- development of entorhinal grid cells and hippocampal place cells to support spatial navigation (Gorchetchnikov and Grossberg, 2007; Grossberg and Pilly, 2012, 2014; Mhatre et al., 2012; Pilly and Grossberg, 2012, 2013a,b, 2014);
- optic flow navigation by the dorsal, or Where. cortical stream (Cameron et al., 1998; Browning et al., 2009a,b; Elder et al., 2009);
- time cells for adaptively timed learning by the hippocampus (Grossberg and Schmajuk, 1989; Fiala et al., 1996; Franklin and Grossberg, 2017);
- analog and place-value numerical representations by the parietal and prefrontal cortices (Grossberg and Repin, 2003);
- auditory streaming (Cohen et al., 1995; Grossberg et al., 2004);
- auditory scene analysis and speaker normalization by the auditory cortex (Cohen et al., 1999; Grossberg et al., 2004; Ames and Grossberg, 2008);
- reinforcement learning by cognitive-emotional interactions within and between multiple brain regions (Grossberg, 1975a, 2018, 2019; Fiala et al., 1996);
- motion vector decomposition due to form-motion interactions across the ventral, or What, and the dorsal, or Where, cortical streams (Grossberg et al., 2011);
- linguistic, spatial, and motor working memories in the prefrontal cortex that can temporarily store event sequences with repeats (Grossberg et al., 1997; Grossberg and Myers, 2000; Grossberg and Pearson, 2008; Grossberg and Kazerounian, 2011, 2016; Silver et al., 2012);
- and sequence categories, or list chunks, in the prefrontal cortex that can encode lists of variable length (Cohen and Grossberg, 1986, 1987; Grossberg and Myers, 2000; Kazerounian and Grossberg, 2014).

Given the number and functional diversity of the types of maps reviewed herein, the model summaries will primarily emphasize the main concepts used in their design. Key references to the broader literature will be included, but the archival articles contain many more.

### Laminar Computing: From Infant Development to Adult Perception, Attention, and Cognition

One important theme describes an emerging computational neural theory of how the laminar circuits of neocortex develop. Indeed, it has long been known that all perceptual and cognitive neocortex seems to have six main layers of cells, in addition to characteristic sublaminae (Martin, 1989; Brodmann, 1909) and that these neocortical circuits integrate bottom-up, top-down, and horizontal interactions. Brodmann (1909) described more than 50 distinct areas of neocortex based on differences in the thickness of the layers, and the sizes and shapes of the neurons within them. How a shared laminar organization might support different behavioral functions of these specialized areas was not, however, clear until a series of articles about Laminar Computing started to explain them (e.g., Grossberg et al., 1997; Grossberg, 1999a; Grossberg and Raizada, 2000; Grossberg and Williamson, 2001).

This theory’s original focus was on the development of the visual cortex. It soon became clear, however, that it has broad implications in other areas of psychology and neuroscience, for at least two reasons. First, emergent properties of the developed circuits simulated psychological and neurobiological data about adult visual perception, attention, and learning, including the basic perceptual processes of 2D and 3D boundary completion and surface filling-in. Second, related modeling studies showed how variations of the same laminar neocortical circuits can help to explain psychological and neurobiological data about adult speech, language, and cognitive information processing, notably about the organization of cognitive and motor working memories and learned sequence categories, also called chunks or plans. These laminar cortical models built upon non-laminar models of brain development that introduced their main design constraints and mechanisms, before additional insights showed how to embody them in laminar cortical circuits with a broader explanatory and predictive range.

Laminar Computing achieves three basic general properties of biological intelligence:

(1) self-stabilizing development and learning;
(2) seamless fusion of pre-attentive, automatic, bottom-up information processing with attentive, task-selective, top-down processing; and
(3) *analog coherence*; namely a solution of the binding problem for perceptual grouping without a loss of analog sensitivity.

In fact, the proposed solution of problem (1) implies solutions to problems (2) and (3). Thus, mechanisms that enable the visual cortex to develop and learn in a stable way impose key properties of adult visual information processing in such a way that there is no strict separation between preattentive processes, such as perceptual grouping, and task-selective attention. A family of models that unifies these themes is called LAMINART because it clarifies how mechanisms of Adaptive Resonance Theory, or ART, which had previously been predicted to occur in neocortex to help stabilize cortical development and learning (e.g., Grossberg, 1980, 1999b), are realized in identified laminar visual cortical circuits (e.g., Grossberg, 1999a). The following text clarifies these issues.

### Feedforward and Feedback: Self-normalizing Circuits Trade Certainty Against Speed

Neocortex can achieve fast feedforward processing when input data are unambiguous (e.g., Thorpe et al., 1996). When multiple ambiguous alternatives exist in the data, processing automatically slows down. This happens because cortical computations are normalized, so that when multiple alternatives exist, each alternative becomes less active, thereby slowing down processing. Intracortical positive feedback loops contrast-enhance and choose the alternative, or alternatives, that are best supported by the data, while negative feedback suppresses weaker alternatives. As the chosen alternatives become more active, their processing speeds up and gives rise to output signals.

Such a system “runs as fast as it can,” trading certainty against speed. Because laminar neocortex uses self-normalizing competition, cell activities can be interpreted as “real-time probabilities” and the process of contrast-enhancement as one of choosing the most likely alternatives. Laminar neocortical dynamics that are modeled by ART go beyond the capabilities of Bayesian decision-making models. Indeed, ART can learn about rare but important events, such as the first outbreak of a disease, for which no priors may exist. ART can do so without confusing the rare event with similar diseases, due to ART’s ability to dynamically regulate the concreteness or abstractness of learned recognition categories using vigilance control (Carpenter and Grossberg, 1987a,b; Grossberg, 2017a). ART can also learn from small and nonstationary databases from which reliable probability estimates cannot be made. It does not need a statistical analysis to succeed.

Talking about statistics: although various ART models do exhibit properties of Bayesian statistics, some go beyond the capabilities of Bayesian classifiers; e.g., Williamson (1996, 1997). Various other ART properties that go beyond Bayesian ones will be described below. More generally, ART circuit designs can be derived from thought experiments whose hypotheses are ubiquitous properties of environments that we all experience (Grossberg, 1980). ART circuits emerge as solutions that satisfy multiple environmental constraints to which humans and other terrestrial animals have successfully adapted. This fact suggests that ART designs may, in some form, have a property of *universality* that may be expected to eventually be embodied in all autonomous adaptive intelligent devices, whether biological or artificial.

### Analog and Digital: Analog Coherence

Analog coherence combines the stability of digital computing with the sensitivity of analog computing. As noted above, making decisions in neural networks typically requires the action of recurrent on-center off-surround networks whose positive on-center feedback helps to choose a winner, while negative off-surround feedback suppresses weaker alternatives. These feedback interactions endow network decisions with useful properties of coherence, notably synchronization and persistence. However, incorrectly designed feedback networks may always allocate maximum activity to a winning cell population, no matter how weak the evidence is for that decision. Such winner-take-all decisions at early stages of processing could undermine the ability of later processing stages to properly weigh accumulating evidence for decision-making. Laminar recurrent on-enter off-surround networks embody the useful properties of coherence, while also allowing grouping strength to increase with the amount of evidence for it.

### Adaptive Resonance Theory and the Stability-Plasticity Dilemma

**Figure 1:**
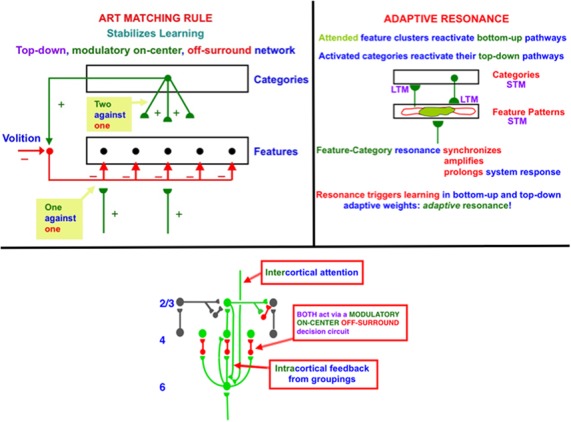
(top row, left column) The ART Matching Rule governs object attention in the brain, notably the ventral, or What, cortical stream. Bottom-up feature signals can, by themselves, activate feature detectors (bottom layer of the figure). A recognition category (top layer of the figure) can activate top-down attentional signals. These top-down signals are carried by a modulatory on-center, off-surround network. The modulatory on-center cannot, by itself, activate its target cells to suprathreshold values, but it can sensitize, or modulate, them in preparation for matching bottom-up signals. The off-surround can, by itself, inhibit its target cells. When bottom-up and top-down signals are both active, then cells that receive two sources of excitatory signals and one source of inhibitory signals can fire (“two against one”), whereas cells in the off-surround are suppressed (“one against one”). (top row, right column) When cells in the on-center of the ART Matching Rule fire, they can reactivate the bottom-up excitatory pathways that originally activated them. An excitatory feedback loop between the feature pattern and category layers is hereby closed, leading to a *feature-category resonance*. (bottom row) A modulatory on-center, off-surround network from layer 6–4 is activated both by intercortical top-down attention and intracortical groupings in layer 2/3. The intercortical pathway supports top-down attention that obeys the ART Matching Rule. This intercortical pathway selects critical features that fall within its modulatory on-center, while inhibiting features that fall within its off-surround. The intracortical pathway enables a grouping to serve as its own attentional prime. It helps to choose a final grouping and to dynamically stabilize its own development. Because the intercortical and intracortical pathways include the same layer 6-to-4 modulatory on-center, off-surround decision network, attentive and preattentive constraints can cooperate and compete to choose the final grouping.

In order to dynamically stabilize learning to prevent catastrophic forgetting, advanced brains use a particular kind of top-down feedback circuit that is said to obey the ART Matching Rule (Figure 1, top row, left column). Without the action of such a feedback circuit, new learning could rapidly erode memories of older learning. This is called the property of *catastrophic forgetting*, a property that is ubiquitous in most neural networks, including backpropagation and the Deep Learning algorithm built upon it. Adaptive Resonance Theory, or ART, which was introduced in 1976 (Grossberg, 1976a,b, 1980) and incrementally developed to the present, is a biological neural network that solves the catastrophic forgetting problem. I prefer to call this problem the *stability-plasticity dilemma* because it requires that fast learning, or *plasticity*, be possible, without also forcing fast forgetting or a loss of memory *stability*. ART solves the stability-plasticity dilemma while overcoming 17 computational problems of backpropagation and Deep Learning (Grossberg, 1988).

Top-down learned expectations and attentional focusing are needed to solve the stability-plasticity dilemma. In particular, the ART Matching Rule governs object attention in the brain (Figure 1, top row, left column). In an ART Matching Rule circuit, bottom-up feature signals can, by themselves, activate feature detectors (bottom layer of the figure). An activated recognition category (top layer of the figure) can, in turn, activate top-down learned expectation signals. The top-down signals define a modulatory on-center, off-surround network. The modulatory on-center cannot, by itself, activate its target cells to suprathreshold values. However, it can sensitize, or modulate, them in preparation for matching bottom-up signals. The off-surround can, by itself, inhibit its target cells. When bottom-up and top-down signals are both active at target cells, then two sources of excitatory signals and one source of inhibitory signals converge upon them, so that they can fire (“two against one”). Cells in the off-surround receive only one source of bottom-up excitatory signals and one source of top-down inhibitory signals, so they are suppressed (“one against one”). An attentional focus hereby forms across the matched cells.

When cells in the on-center of the ART Matching Rule fire, they can reactivate the bottom-up excitatory pathways that originally activated them. An excitatory feedback loop between the feature pattern and category layers is hereby closed. It triggers a *feature-category resonance* that synchronizes, amplifies, and prolongs system activity, focuses attention upon the feature pattern that is resonating, and supports conscious recognition of the resonating category and its feature pattern (Figure 1, top row, right column; Grossberg, 2017b). Such a resonance can trigger learning in the adaptive weights in active bottom-up pathways and top-down expectation pathways.

Thus, unlike artificial neural networks like backpropagation and Deep Learning that include only feedforward, or bottom-up connections, a biological theory like ART includes bottom-up and top-down connections in order to solve the stability-plasticity dilemma. ART also includes recurrent horizontal connections to choose the categories whose top-down expectations are matched against bottom-up signals.

ART is currently the most advanced cognitive and neural theory about how brains learn to attend, recognize, and predict objects and events in a changing world that includes unexpected events. This claim is supported by the fact that ART has explained and predicted more psychological and neurobiological data than other theories of how brains learn, and all the computational hypotheses upon which ART is based have been supported by subsequent psychological and neurobiological data. See Grossberg (2013, 2017a,b, 2018, 2019, 2020) for reviews.

### Preattentive and Attentive Learning

In the LAMINART and 3D LAMINART models that develop ART to include cortical layers and identified cortical cells within them, both *inter*cortical and *intra*cortical feedback circuits obey the ART Matching Rule (e.g., Grossberg and Raizada, 2000). In particular, both intercortical and intracortical pathways share a key decision circuit in the deeper layers, between layers 6 and 4, of each cortical area (Figure 1, bottom row). In particular, the *inter*cortical circuits realize top-down attention, which can dynamically stabilize learning using a modulatory on-center, off-surround network from a higher cortical region to a lower one. For example, layer 6 in the cortical area V2 can attentionally prime processing in V1 *via* a circuit of this type. Here active cells in layer 6 in V2 send excitatory topographic signals to cells in layer 6 of V1, either directly or *via* layer 5 cells. The activated V1 cells, in turn, send signals to layer 4 in V1 *via* modulatory on-center, off-surround interactions. Taken together, these various signals realize an *intercortical, top-down, modulatory on-center, off-surround network*. The flow of signals from layer 6-to-6 and then back from layer 6-to-4 is said to embody *folded feedback*.

*Intra*cortical circuits help to dynamically stabilize the development of long-range horizontal connections that form *via* recurrent signals among cells in layer 2/3. In addition to these intralaminar recurrent interactions, interlaminar but intracortical signals help to stabilize development and learning among the layer 2/3 neurons. In particular, cells in layer 2/3 of V2 send excitatory signals to cells in layer 6 of V2. The activated V2 cells, in turn, send signals to layer 4 in V2 *via* modulatory on-center, off-surround interactions. This is the same example of folded feedback that realizes top-down attention. Here, however, it occurs within a cortical area in response to activation of horizontal cortical groupings that can form automatically and preattentively, or in the absence of attention.

Perceptual groupings are completions of boundaries in the interblob cortical stream. They include illusory contours, as well as groupings of 2D shading gradients and texture elements that support filling-in of brightnesses and colors to create 3D surface representations (Grossberg and Pessoa, 1998; Kelly and Grossberg, 2000). To emphasize the difference between intercortical and intracortical forms of attention, I like to say that “a preattentive grouping is its own attentional prime.”

In summary, both the intercortical and intracortical circuits include the same layer 6-to-4 modulatory on-center, off-surround network of interactions. This shared network is said to be an *attention-preattention interface*. It is here that contextual constraints of preattentive grouping and task-related top-down attention come together to decide which from the set of possible groupings will be chosen in the current visual context.

## ART Matching Rule Solves Stability-Plasticity Dilemma *via* Attention-Preattention Interface

As noted above, Adaptive Resonance Theory, or ART, uses top-down attention that obeys the ART Matching Rule to enable advanced brains to solve the *stability-plasticity dilemma*, whereby our brains can rapidly learn throughout life, without also rapidly forgetting what they already know. Rapid brain *plasticity* can thus occur without losing the memory *stability* that prevents catastrophic forgetting. The generality of the stability-plasticity dilemma suggests that similar top-down mechanisms should occur between multiple cortical areas wherein self-stabilizing learning can occur.

What circuits does top-down attention modulate? Answering this question leads to the assertion that “a preattentive grouping is its own attentional prime,” as well as to an understanding of how solving problem (1) above also solves problems (2) and (3). This is because one of the most important types of circuits that top-down attention modulates during vision is the perceptual groupings that form due to interactions among long-range horizontal connections in layer 2/3. With perceptual groupings in mind, it can readily be seen that an improper solution to the stability-plasticity problem could easily lead to an *infinite regress*, because perceptual groupings can form automatically and preattentively before providing a neural substrate upon which higher-level attentional processes can act. But how can a preattentive grouping develop in a stable way, before the higher-order attentional processes can develop with which to stabilize them? In particular, how can long-range horizontal connections in layer 2/3 of cortical area V1 develop before they can be modulated by top-down attention from cortical area V2? If such preattentive mechanisms cannot deliver reliable signals to the higher cortical areas, then any top-down signals from these higher areas may be of little use in stabilizing their own development.

I called this the *attention-preattention interface problem* because the laminar cortical circuits include layers (the *interface*) where both preattentive and attentive mechanisms can come together, notably layers 6-to-4 in Figure 1 (bottom row), to help determine which of several possible “preattentive” groupings will be chosen.

The existence of this kind of cortical interface within multiple intercortical and intracortical feedback loops clarifies why distinguished scientists have debated for decades about the distinction between preattentive and attentive processes, as illustrated by some descendants of the great vision scientists Hermann von Helmholtz (von Helmholtz, 1866, 1962), who emphasized top-down interactions that were a precursor of current Bayesian concepts, and Gaetano Kanizsa (Kanizsa, 1955, 1974, 1976), who emphasized bottom-up and horizontal interactions. As illustrated by Figure 1 (bottom row), all three types of processes—bottom-up, horizontal, and top-down—interact strongly using shared decision circuits within the attention-preattention interface.

## Why Does Not the Development of Preattentive Groupings Violate the ART Matching Rule?

The fact that “a preattentive grouping is its own attentional prime” solves a challenging problem for perceptual groupings, such as illusory contours, that can generate suprathreshold responses over positions that do *not* receive bottom-up inputs. They, therefore, seem to violate the ART Matching Rule, which asserts that, in order for cortical learning to be stable, only cells that get bottom-up activation should be able to fire to suprathreshold levels. That is one reason why circuits that embody the ART Matching Rule can only modulate the activities of the cells in their on-centers. How, then, can the horizontal connections that generate perceptual groupings maintain themselves in a stable way? Why are they not washed away whenever an illusory contour forms across positions that do not receive a bottom-up input? The answer is now clear: At *every* position where an illusory contour forms, the preattentive grouping is its own attentional prime, so that development and learning at that position are dynamically stabilized by the same modulatory on-center, an off-surround circuit that attention can use to stabilize learning. The current analysis hereby proposes an answer to this question that clarifies how perceptual grouping, attention, development, and adult perceptual learning are intimately bound together within the laminar circuits of the visual cortex.

## Infant Development and Adult Learning Use Similar Laws: A Universal Developmental Code

This conclusion illustrates an even broader generalization: both psychological and neurobiological data support the idea that the neural laws that regulate infant development and adult learning in grouping and attentional circuits are the same. Supportive data include the fact that the horizontal connections that support perceptual grouping in cortical areas like V1 and V2 develop through a learning process that is influenced by visual experience (Callaway and Katz, 1990; Löwel and Singer, 1992; Antonini and Stryker, 1993). It is also known that many developmental and learning processes, including those that control horizontal cortical connections, are stabilized dynamically, and can be reactivated by lesions and other sources of cortical imbalance (Gilbert and Wiesel, 1992; Das and Gilbert, 1995) in order to relearn the environmental statistics to which the new cortical substrate is exposed.

More generally, adult learning often seems to use the same types of mechanisms as the infant developmental processes upon which it builds (Kandel and O’Dell, 1992). This was one of the guiding themes behind early ART predictions from the 1970s about how brain circuits that form during infant development can support later learning that refines and builds upon them. For example, two articles that were published back-to-back in the 1978 annual volume of *Progress in Theoretical Biology* developed this theme. One article is called Communication, Memory, and Development (Grossberg, 1978b), a title that underscores the article’s proposal that all cellular tissues, both inside and outside brains, embody a universal developmental code whose mathematical laws are often formally the same as those that control later learning, albeit possibly realized by different physical mechanisms; e.g., directed growth of connections during development vs. learned tuning of synaptic connections during adult learning. The other article is called A Theory of Human Memory: Self-organization and Performance of Sensory-motor Codes, Maps, and Plans (Grossberg, 1978a), a title that summarizes the article’s goal of discussing various learning processes that occur after infant development. This article included contributions to ART. Both articles laid theoretical foundations for many additional model developments during the subsequent decades.

## Laminart Circuits for Development and Adult Preattentive Grouping and Attention

**Figure 2:**
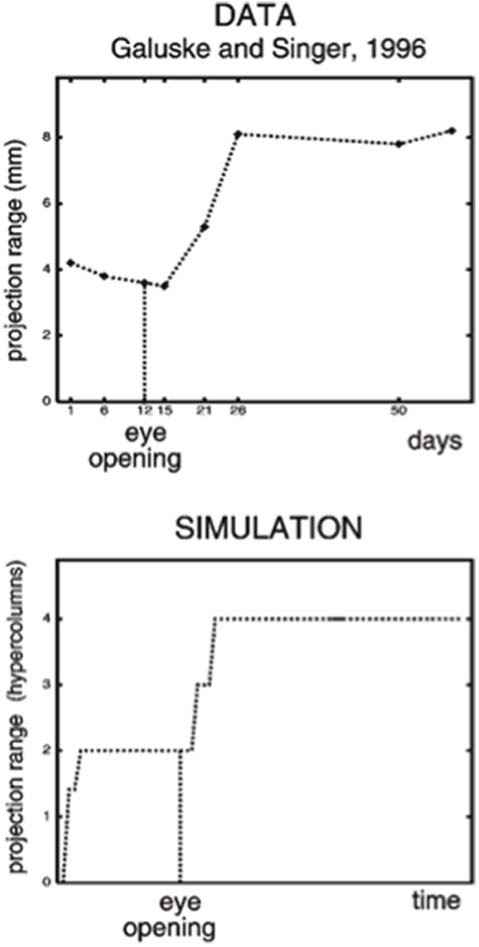
(Top image) The projection range of pyramidal cells in cat visual cortex doubles after eye opening [adapted from Galuske and Singer (1996)]. (Bottom image) The same thing happens during model development [reprinted with permission from Grossberg and Williamson (2001)].

The LAMINART model proposes how horizontal and interlaminar connections develop in cortical areas V1 and V2, after which they support adult perceptual grouping and attention. During the development of perceptual groupings, initially there is crude clustering of weak horizontal connections until patterned visual input occurs after eye opening. Visual input strengthens and refines these connections while doubling the projection range of long-range horizontal connections in layer 2/3 (Figure 2, top image). These horizontal connections also double their length in the model’s layer 2/3 (Figure 2, bottom image) between iso-orientation columns, preferentially along each cell’s preferred orientation, leading to cells that exhibit other experimentally reported properties, such as length summation and appropriate responses to surround stimuli, as well as the temporal sequencing and relative amounts by which different V1 laminae develop orientation selectivity.

**Figure 3:**
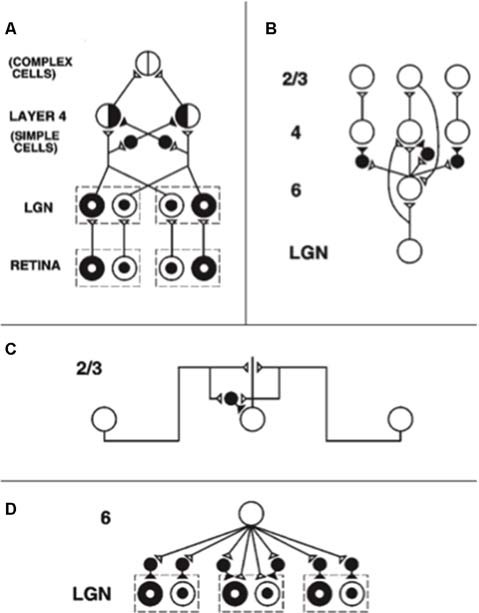
The adult network of retinal, lateral geniculate nucleus (LGN), and cortical V1 neurons to which the developmental model converges. **(A)** Feedforward circuit from the retina to LGN to cortical layer 4: retinal ON cells have an on-center off-surround organization (white disk surrounded by black annulus). Retinal OFF cells have an off-center on-surround organization (black disk surrounded by white annulus). LGN ON and OFF cells receive feedforward ON and OFF cell inputs from the retina, which activate excitatory inputs to layer 4 oriented simple cell receptive fields. Like-oriented layer 4 simple cells with opposite contrast polarities compete before generating half-wave rectified outputs that are pooled at complex cells, which can thus respond to both polarities. **(B)** Cortical feedback loop between layers 6, 4, and 2/3: LGN activates layer 6 and layer 4 cells. Layer 6 cells excite layer 4 cells with a narrow on-center and inhibit them using layer 4 inhibitory interneurons within a broader off-surround. Layer 4 cells then excite layer 2/3 cells, which send excitatory feedback signals back to layer 6 cells *via* layer 5 (not shown). Layer 2/3 can hereby activate the layer 6-to-4 modulatory on-center, off-surround network. **(C)** Horizontal interactions in layer 2/3 support perceptual grouping: Layer 2/3 complex pyramidal cells monosynaptically excite one another *via* horizontal connections, primarily on their apical dendrites, and inhibit one another *via* disynaptic inhibition *via* model smooth stellate cells. **(D)** Top-down corticogeniculate feedback from layer 6: Layer 6 cells send topographic excitatory signals to LGN ON and OFF cells and broadly distributed inhibitory signals *via* LGN inhibitory interneurons. The feedback signals pool outputs over all cortical orientations and are delivered equally to ON and OFF cells [reprinted with permission from Grossberg and Williamson (2001)].

During development, random or visual inputs from lateral geniculate nucleus (LGN) excite cells in layer 4 (Figure 3A) which in turn activate cells in layer 2/3 (Figure 3B), where the horizontal connections self-organize between cells responding to different orientations and locations according to correlational and competitive growth rules (Figure 3C). This developmental process results in a network of long-range horizontal excitatory connections between layer 2/3 model pyramidal cells, along with shorter-range disynaptic inhibitory connections mediated by layer 2/3 model smooth stellate cells.

Such a network supports inward perceptual grouping between two or more approximately collinear and like-oriented boundary inducers, but not outward grouping from a single inducer. This property is called the *bipole grouping rule*. Form-sensitive scenic boundaries are hereby completed. The existence of bipole cells was predicted in Grossberg (1984) and simulated in a series of articles beginning with Grossberg and Mingolla (1985). The first neurophysiological evidence for bipole cells was reported in cortical area V2 by von der Heydt et al. (1984).

As noted above, boundary signals from layer 2/3 feedback to layer 4 *via* layer 6, which sends on-center excitation and adaptive off-surround inhibition to layer 4 (Figure 3C). The model develops V2 connections using similar rules, but with larger spatial scales.

## Balanced Excitation and Inhibition Enable Both Grouping and Attention Circuits to Develop

The LAMINART model clarifies how the excitatory and inhibitory connections that occur in these circuits can develop by maintaining a balance between excitation and inhibition. The growth of long-range excitatory horizontal connections between layer 2/3 pyramidal cells is balanced against that of short-range disynaptic interneuronal connections. Within the attention-preattention interface that is shared by both grouping and attentional pathways, the growth of excitatory on-center connections from layer 6-to-4 is balanced against that of inhibitory interneuronal off-surround connections.

**Figure 4:**
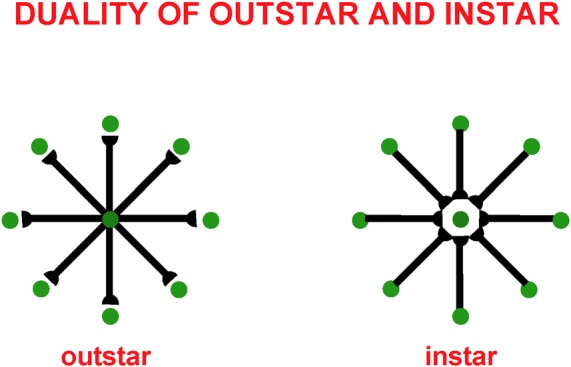
Instar and outstar networks can both learn spatial patterns of activity from the network of cells that they sample. When the source cell of an outstar is active, its adaptive weights sample and learn a time-average of the activities in the outstar’s border cells. The outstar can then read-out the net spatial pattern of activity that it learned across the border cells. When the source cell of an instar is active, its abutting adaptive weights learn a time-average of the axonal signals that they gate from sampled cells. This tuning process makes the source cell fire more selectively to activity patterns across the sampled cells that match it. The source cell functions like a recognition category for that, and similar, activity patterns.

These balanced connections have been shown through theorems and computer simulations in Grossberg and Williamson (2001) to develop properly using a combination of outstar (Grossberg, 1968, 1971) and instar [Grossberg, 1976a, 1980 (Appendix)] learning laws. The names of these learning laws reflect the anatomies in which they occur (Figure 4). In an outstar, when a “source cell” in the center of the outstar (green disk) is activated, it sends a sampling signal along all of its axons to the synapses at their ends, which are drawn in Figure 4 as hemidisks. In these synapses, adaptive weights, or long term memory (LTM) traces, begin learning whenever a sampling signal is active. The LTM traces can increase to match large postsynaptic activities or decrease to match small ones. Through time, these LTM traces learn a time-average of the activities of the cells that they abut whenever their sampling signal is active. In this way, an outstar can learn a time-averaged *spatial pattern* of activities of the cells in its border. In different specialized circuits, these spatial patterns can represent a wide range of specific patterns, ranging from top-down cognitive expectations to motor synergies.

The anatomy of an instar differs from that of an outstar by reversing the direction of the signal flow in its axons. This is the *duality* property of instar and outstar in Figure 4. Thus, in an instar, the cell that triggers learning receives signals from all the cells in the instar border. When it is activated, this cell triggers learning in all LTM traces within its abutting synapses. The pattern, or vector, of all these LTM traces, hereby becomes more parallel to the time-averaged pattern of all the input signals that they experience when the sampling cell is active. After learning occurs, input patterns that are more parallel to the LTM vector more vigorously activate their shared postsynaptic cell.

This tuning process supports the learning of recognition categories in self-organizing maps (SOM) and ART networks, among many others. In such networks, multiple sampling cells compete. The cells that are almost parallel to the current input pattern have the highest activation and win the competition. In this way, such a network’s input patterns selectively activate the recognition categories that best represent them.

The development of grouping and attentional circuits in laminar neocortical networks is yet another of the applications where outstar and instar learning are valuable. *Instar* learning helps to tune the growth and selectivity of excitatory horizontal connections in layer 2/3, whereas *outstar* learning helps to tune how inhibitory interneurons balance excitation in layers 4 and 2/3 (Grossberg and Williamson, 2001).

Outstars and instars are typically used in rate-based neural networks. There has been substantial progress since their introduction in showing how their activation and learning laws can be embedded in spiking networks with detailed biophysical and biochemical interpretations, including a method for transforming any rate model that uses membrane equations into an equivalent spiking model (e.g., Fiala et al., 1996; Cao and Grossberg, 2012; Pilly and Grossberg, 2013b). This rich theme of work on learning will not be further discussed herein.

## Balanced Signals Support Sparse and Variable Spiking as Well as Rapid Synchronization

The balance between excitatory and inhibitory interactions helps to explain the observed sparseness and variability in the number and temporal distribution of spikes emitted by cortical neurons (Shadlen and Newsome, 1998; van Vreeswijk and Sompolinsky, 1998). This kind of spiking does not efficiently activate neurons, but may provide background activation that helps to maintain homeostatic plasticity during periods of rest (Turrigiano, 1999). Given this inefficiency, how do neurons ever fire efficiently? A property of such balanced networks, at least when they are properly designed using neuronal membrane equations that include automatic gain control by shunting interactions (e.g., Grossberg, 2013), is that, when they are driven with external inputs, their activities are rapidly amplified and synchronized, thereby achieving efficient processing (e.g., Grossberg and Williamson, 2001; Grossberg and Versace, 2008).

The LAMINART model hereby suggests that a balance between excitation and inhibition in multiple cortical layers ensures several useful properties: stable development and learning by cortical circuits, perceptual grouping and attention, a baseline of inputs during resting states to support homeostatic plasticity, and rapid, efficient, and synchronous processing of input patterns during performance.

Various other authors have also emphasized a role for balanced excitation and inhibition, including in the development of map properties such as orientation tuning in primary visual cortex (Mariño et al., 2005) and frequency tuning in primary auditory cortex (Sun et al., 2010).

## How Does the Cortical Map Develop in the Laminar Cortex of Cortical Area V1?

The above results do not yet explain how the development of cortical maps in cortical area V1 may occur, how this development may be coordinated across cortical layers to form cortical columns (Hubel and Wiesel, 1974), or how maps form that coordinate inputs from both eyes. In particular, in cortical area V1, cells tuned to orientation and ocular dominance are found within its map (Blasdel, 1992a,b; Crair et al., 1997a,b; Hübener et al., 1997). The V1 map is, however, only one of many in the brain. Topographically organized maps in functional columns have been found in visual (Tootell et al., 1982, 1998; Duffy et al., 1998), auditory (Komiya and Eggermont, 2000; Stanton and Harrison, 2000), somatosensory (Dykes et al., 1980; Grinvald et al., 1986; Wallace and Stein, 1996) and motor (Nieoullon and Rispal-Padel, 1976; Munoz et al., 1991; Chakrabarty and Martin, 2000) thalamic and cortical areas. An important task in understanding the brain, and in building computational models thereof, is to explain the organizational principles and mechanisms whereby such maps develop and are coordinated between interacting cortical columns. The cortical map in V1 will be discussed first as a prototype for maps in other modalities.

Early neural models proposed how maps of orientation (OR), ocular dominance (OD), and related properties may develop in V1 (e.g., von der Malsburg, 1973; Grossberg, 1975b, 1976a; Willshaw and von der Malsburg, 1976; Swindale, 1980, 1992; Kohonen, 1982; Linsker, 1986a,b,c; Miller et al., 1989; Rojer and Schwartz, 1990; Olson and Grossberg, 1998). These models showed how the spontaneous activity that occurs before eye opening, when it interacts with associative learning and competitive interactions, can generate maps with properties similar to those found *in vivo*. However, these results did not explain how cortical columns develop the consistent tuning for orientation and ocular dominance that is observed along with vertical penetrations across multiple cortical layers (Hubel and Wiesel, 1974). This was a significant challenge for models because the orientation maps in layers 4 and 6, as well as the crude clustering in layers 2/3 and 5, begin to develop before interlaminar connections within V1 exist with which to coordinate their formation across layers (Callaway and Katz, 1992). These initial preferences are, moreover, preserved and refined in response to patterned vision after eye opening (Callaway and Katz, 1990, 1991). It was thus an urgent question to explain how these initially shared properties across cortical layers could be coordinated without interlaminar connections that arise within the cortex itself.

Grossberg and Seitz (2003) proposed that this coordination is realized by the cortical subplate (Rakic, 1976; Luskin and Shatz, 1985; Allendoerfer and Shatz, 1994; Ghosh and Shatz, 1994; Ghosh, 1995; McAllister, 1999). The subplate exists transiently as a kind of extra deep layer of V1 where it receives thalamocortical connections at an early stage of development. These connections wait for weeks before innervating layer 4. During that time, the subplate sends vertical connections throughout the developing cortical plate (Ghosh and Shatz, 1993; McConnell et al., 1994). The critical developmental role of the subplate was also illustrated by the fact that its ablation prevents the formation of cortical cells tuned to orientation (Kanold et al., 2003) and ocular dominance maps (Ghosh and Shatz, 1992).

Grossberg and Seitz (2003) modeled how a cortical map develops within the subplate, and sends signals topographically through the cortical layers. These topographic subplate signals act as teaching signals whereby the early consistent tuning for orientation and ocular dominance across layers is achieved, even before interlaminar connections within V1 exist. The subplate’s interlaminar topographic signals also activate the growth of topographic interlaminar pathways within the cortex that support cortical columns.

## Why Is the Subplate Needed for Cortical Development?

This overview raises the question of why the subplate is needed, given that there are successful models of cortical map development that do not require a subplate. Grossberg and Seitz (2003) proposed that the subplate ensures the development of topographically precise cortical columns that coordinate the activities of cells in multiple cortical layers, as in Figure 1 (bottom row). The subplate avoids a serious problem that was shown to occur in a laminar model of cortical development without a subplate. Without a subplate to guide the topographic growth of interlaminar connections, long-range horizontal connections in layer 2/3, among others, caused correlations across multiple cortical positions, resulting in interlaminar connections distributed broadly across the network, rather than in topographical cortical columns. A major reduction in the spatial resolution of cortical representations was hereby caused. Such a cortex could not represent the orientations and eye of origin from a sufficient number of retinal positions to provide adequate visual acuity using cortical maps.

The subplate hereby resolves a design tension between the need to provide adequate visual acuity using topographic cortical columns that can learn to become selective to different visual features, and the need to enable long-range horizontal processes like perceptual grouping to occur. Because earlier models of V1 map development included neither cortical layers nor long-range cortical interactions, this problem did not occur in them.

## STM, MTM, and LTM in Cortical Map Development

As in earlier models of cortical development, the Grossberg and Seitz (2003) subplate model proposed that subplate circuits embody a source of noisy input, a bandpass filter, and normalization across model cells. Moreover, all model cortical layers realize bandpass filter and normalization properties, which arise naturally in the networks of on-center off-surround interactions between cells that obey membrane, or shunting, equations (Grossberg, 1973, 1976a, 1980, 2013). As noted above, such networks can balance cell cooperation and competition and thereby enable network neurons to remain sensitive to the relative size of inputs whose total size may vary greatly through time.

When these networks include recurrent interactions, they can also contrast enhance their cell responses to input patterns while normalizing them. In particular, contrast enhancement amplifies cell activities in response to their small initial inputs due to the small size of bottom-up adaptive weights before development occurs. The contrast-enhanced activities enable development to occur efficiently by helping to choose the cell population, or small set of populations, that receive the largest inputs. These winning cells can then drive instar learning in the LTM traces within the synapses that abut them, and thereby tune the adaptive filters that learn the cortical map.

The above comments invoke short term memory (STM) traces, or cell activations, as well as LTM traces, or adaptive weights, in map development. No less important are medium-term memory (MTM) traces, or habituative transmitter gates (Grossberg, 1976a, 1980), which also occur in so-called depressing synapses (Abbott et al., 1997) and dynamic synapses (Tsodyks et al., 1998). MTM traces occur in the subplate and in the subsequently developing cortical layers. These transmitters gate, or multiply, the axonally-mediated signals between cells and habituate in an activity-dependent way.

MTM traces overcome a serious problem that could impede development in their absence; namely, they prevent the cells that first win the competition from persistently dominating network dynamics thereafter, due to the fact that their LTM traces have become larger. Because the STM signal in a pathway is multiplied both by LTM *and* MTM traces before a net signal activates target cells, the increasing size of the LTM trace can be offset by the decreasing size of the corresponding MTM trace when a given STM signal has been active for a while. After the MTM traces recover, the larger LTM traces can again help to choose winning cells in response to input patterns that their LTM vector best matches.

## Temporal Organization of Stages in Cortical Map Development

### Spontaneous Retinal Waves Drive the Development of Retina-to-LGN and LGN-to-Subplate Connections

**Figure 5:**
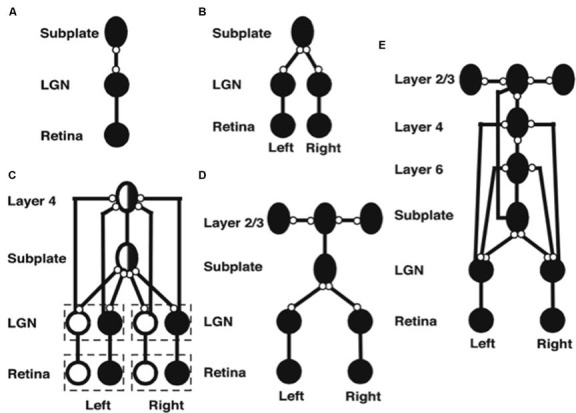
Stages of model development (black circles denote OFF receptive fields, white circles denote ON receptive fields, ovals denote orientationally tuned cells, lines ending in open circles denote adaptive connections, lines ending without circles denote non-adaptive connections). **(A)** Monocular subplate circuit: spontaneous activity in retinal OFF cells activates the LGN, which in turn activates the subplate. Feedforward adaptive weights from the LGN to the subplate and feedback adaptive weights from the subplate to the LGN develop a map of oriented receptive fields. **(B)** Binocular subplate circuit: inputs from the second eye activate, leading to learning of a map of ocular dominance in the subplate that is superimposed on the existing orientation map. **(C)** Binocular layer 4 circuit: the ocular dominance and orientation maps in the subplate are taught to layer 4. Retinal ganglion ON cells activate and correlated retinal inputs help to segregate ON and OFF subfields in layer 4. **(D)** Layer 2/3 circuit: clusters of horizontal connections develop in layer 2/3, driven by the correlations in subplate inputs. **(E)** Fully developed model: Layer 6 develops connections to and from the LGN. Then interlaminar connections develop from layer 6 to layer 4, and from layer 4 to layer 2/3. After the subplate and its connections are removed, model maps remain stable [reprinted with permission from Grossberg and Seitz (2003)].

The model’s initial circuit contains the retina, LGN and subplate (Figure 5A). Several types of data support the hypothesis that this circuit is monocular: Neurophysiological recordings in area 17 of kittens show that, at eye opening, the majority of cells respond only to contralateral eye inputs (Albus and Wolf, 1984). In young ferrets, LGN activity is largely unchanged when ipsilateral inputs from the retina are cut (Weliky and Katz, 1997). In addition, there exists an early bias of oriented OFF cell activity in the retina (Wong and Oakley, 1996) and the kitten cortex (Albus and Wolf, 1984) before eye opening. Accordingly, the model contains only OFF ganglion cells at this stage of development.

Spontaneous activity arising in the retina drives the development of model feedforward and feedback connections between the LGN and the subplate. After development, the pattern of feedforward connections to a given subplate cell and the feedback connections from that cell share the same axis of elongation (Murphy et al., 1999).

Markowitz et al. (2012) have additionally modeled how spontaneous retinal activity in the form of retinal waves can drive retinogeniculate map development before the LGN connects to the subplate. This study simulates how suitably defined retinal waves guide the connections from each eye into distinct LGN layers A and A1, while these connections also develop in topographic registration. The details of this model can be found in the article.

### Development of Ocular Dominance Columns

The next steps in model development clarify how pathways from both eyes are coordinated during development to form ocular dominance columns in the subplate (Figure 5B). First, connections from the contralateral eye develop a monocular cortical map with orientation columns to the subplate. Activity from the ipsilateral eye begins subsequently, and uses the scaffold of the contralateral eye map, abetted by interocular correlated activity due to processing of the same visual inputs, to create a binocular map with ocular dominance columns. In this way, the ipsilateral eye inherits the orientation map of the contralateral eye, just as receptive fields of the cortical layers will subsequently inherit properties of the subplate.

### A Subplate Map Is Taught to the Other Cortical Layers

After the subplate forms, it guides map formation in the cortical layers. In the model, each cortical layer develops separately. This property is consistent with the fact that, *in vivo*, learning in layer 2/3 occurs after layer 4 has developed its orientation map (Callaway and Katz, 1992; Galuske and Singer, 1996). The development of layer 4 is guided by topographic afferents from the subplate as afferents from the LGN begin to develop into layer 4 (Figure 5C). The endogenous retinal activity enables layer 4 inputs from the subplate to teach developing connections from the LGN into layer 4. The layer 4 LTM traces stabilize as a map similar to that found in the subplate is learned. Maps of ocular dominance and orientation tuning also form in layer 6 (Figure 5E) at a time and manner similar to the developing map in layer 4, as will be explained more fully below.

Development of the horizontal connections in layer 2/3 (Figure 5D) begins when subplate inputs reach this layer. *in vivo*, these subplate inputs are carried by axons that terminate in the marginal zone (Ghosh, 1995) where layer 2/3 cell dendrites occur (Callaway, 1998). In the model, long-range horizontal connections between layer 2/3 cells develop in response to lateral correlations within the subplate inputs. Recurrent signals within these developing layer 2/3 connections amplify the subplate-activated correlations, leading to refinement of the specificity of connections. The subplate inputs to layer 2/3 are the same as those to layer 4, but in layer 2/3 lateral connections develop instead of connections from the LGN. The horizontal connections in layer 5 are proposed to develop in a similar manner to those of layer 2/3.

### Development of Interlaminar Connections

After maps develop in each of the cortical layers, interlaminar connections grow (Callaway and Katz, 1992). In the model, layer 6-to-4 and layer 4-to-2/3 connections develop (Figure 5E). They do so vertically through the cortical layers because the subplate provides the same teaching input to each of them. These vertical interlaminar connections support the dynamics of adult cortical columns. Poorer correlations between cortical layers developed in the absence of subplate teaching signals.

### Development of the Layer 6 Map and Subplate Atrophy

Layer 6 develops a map from the LGN that is topographically similar to the map in layer 4 using the same subplate inputs as layer 4 does. Layer 6 also develops a set of top-down connections to the LGN (Figures 3D, 5E), which are similar to those from the subplate to the LGN (Figures 5D,E).

The subplate is a transient structure that atrophies after the cortical maps and interlaminar cortical connections form. In the model, after the layer 6 connections form, the subplate is removed. Simulations without the subplate demonstrate that the developed cortical architecture is stable. Grossberg and Seitz (2003) also simulated more subtle factors that influence cortical development, such as the role of BDNF (Ghosh and Shatz, 1994; Cabelli et al., 1995, 1997; Berardi and Maffei, 1999).

### Development of ON and OFF Regions in Simple Cell Receptive Fields

As noted above, during early development, oriented cells found in the cortex are monocular and dominated by OFF inputs. During normal development, layer 4 simple cells quickly develop distinct ON and OFF input fields (Albus and Wolf, 1984). In contrast, dark rearing of the ferret causes convergence of ON and OFF signals to LGN cells (Akerman et al., 2002). How distinct but spatially correlated ON and OFF subfields develop is clarified by their properties in response to visual inputs (Schiller, 1992). After the eyes open, the mean firing rates of ON and OFF retinal cells equalizes. Moreover, their activities become anti-correlated because, when an ON cell is active, the OFF cell at that location is hyperpolarized and spatially neighboring OFF cells are active, due to the organization of these cells in on-center off-surround networks within each cell type, and opponent, or competitive, interactions between cell types at each position. Such a network is called a *double opponent* network. These anti-correlated activities across position help to drive selective learning of ON cell inputs to the ON subfield of a simple cell’s receptive field, and OFF cell inputs to the OFF subfield of the cell (Figure 5C).

**Figure 6:**
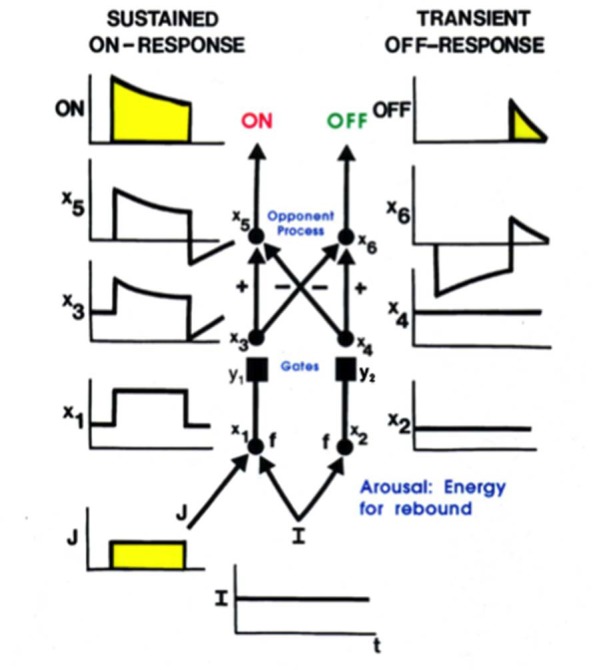
A gated dipole opponent network can generate a transient antagonistic rebound from its OFF channel in response to offset of a sustained input (J) to its ON channel. This can happen because a nonspecific, tonically active, arousal input (I) equally energizes both the ON and OFF channels. The phasic input J to the ON channel habituates its transmitter gate (y_1_) and thus its net output after competition occurs between the ON and OFF channels (see sustained ON-response in yellow). When J shuts off, the net input to the OFF channel is larger than that to the ON channel, because both ON and OFF channels are now driven by the same arousal input I, but the ON channel gate (y_1_) is more habituated than the OFF channel gate (y_2_). An antagonistic rebound then occurs in the OFF channel. The rebound is transient (see transient OFF-response in yellow) because it gradually causes an equal amount of habituation to occur in the transmitter gates of both the ON and OFF channels.

Additional properties are needed to explain how, at each position, a pair of simple cells with oppositely polarized ON and OFF subfields develop. An answer can be found in the properties of the LGN double opponent networks of ON cells and OFF cells. In addition to their fast STM interactions, such networks also include MTM habituative transmitter gates in the opponent circuits whereby ON and OFF cells interact at each position. Such a network is called a *gated dipole field* (Grossberg, 1980). Its individual ON and OFF opponent cells at each position are said to form a *gated dipole*. Grossberg (1972a,b) showed how the offset of a sustained input to an ON cell in a gated dipole can cause an antagonistic rebound that transiently activates the corresponding OFF cell (Figure 6).

Grunewald and Grossberg (1998) have simulated the dynamics of antagonistic rebounds between LGN ON and OFF cells that interact within gated dipole fields during the development of simple cells that are also organized into ON and OFF cell pairs. Suppose that a visual input has activated ON and OFF inputs to an LGN cell, which in turn activates a cortical cell whose orientation and contrast polarity begin to develop into a simple cell with the same orientation and contrast polarity preference. When that visual input turns off, its developing simple cell also shuts off. As a result, antagonistic rebounds in the LGN ON and OFF cells cause LGN OFF and ON cells to transiently turn on, at the same time as the opponent cell of the developing simple cell also turns on. This opponent cell can then begin to learn how to become a simple cell, but one that responds to an opposite polarity input with the same orientation in the same position. When such an opposite polarity input later turns on at this position, this prior partial development give the opposite polarity cell an advantage in winning the competition with other cells. Its development as an opposite polarity cell with the same orientation and position can hereby continue. In this way, opponent pairs of simple cells with like orientation and opposite contrast polarity selectivity can develop in the same position.

### Development of Complex Cells: How Anti-correlated Simple Cells Input to a Complex Cell

The development of opposite polarity simple cells with the same orientation preference at each position sets the stage for the development of complex cells. Complex cells pool inputs from pairs of like-oriented simple cells at the same position but with opposite contrast polarities; that is, with ON-OFF and OFF-ON receptive subfields across position (Hubel and Wiesel, 1962; Movshon et al., 1978). As a result, when one of the simple cells that inputs to a complex cell is active, the simple cell with the opposite contrast polarity preference at that position is silent. Their activities are anti-correlated. How does a complex cell learn to get activated by pairs of anti-correlated simple cells?

An answer follows from the previous discussion of how opposite polarity simple cells develop. In particular, suppose that a simple cell with a given contrast polarity is activated, and starts learning to activate a complex cell. When that simple cell turns off, its opposite polarity simple cell turns on due to a rapid antagonistic rebound. If the previously activated complex cell stays active during this rebound period, it can begin to become correlated with the simple cells of both contrast polarity preferences. Then, just as in the development of opposite polarity simple cells, this initial advantage of the opposite polarity simple cell in activating the complex cell will give it a competitive advantage in response to later inputs that directly turn it on, so that its opposite polarity learning can continue. Grunewald and Grossberg (1998) have simulated the development of complex cells using this kind of dynamics. That article, as well as Grossberg and Grunewald (2002), also simulates how these complex cells develop with a prescribed binocular disparity preference, which is known to occur *in vivo* (Ohzawa et al., 1990). They also simulated development of the top-down connections from complex cells in V1 to the LGN that carry out a matching process *via* the ART Matching Rule, which dynamically stabilizes both bottom-up and top-down learning (Varela and Singer, 1987; Sillito et al., 1994).

## 3D Laminart: Binocular Visual Processing by Laminar Cortical Circuits

**Figure 7:**
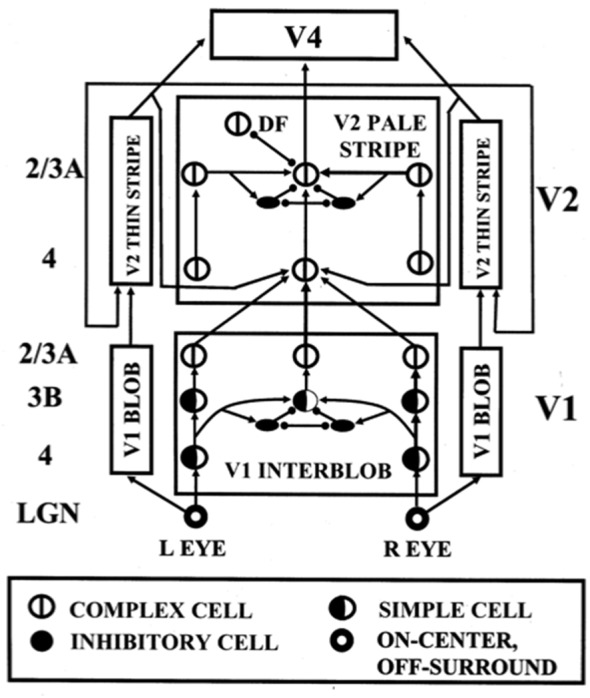
The 3D LAMINART model circuit diagram. The model consists of a (V1 Interblob)—(V2 Pale Stripe)—V4 boundary stream which computes 3D perceptual groupings, and a (V1 Blob)—(V2 Thin Stripe)—V4 surface stream which computes 3D surface representations of lightness, color, and depth. The two processing streams interact to overcome their complementary deficiencies (Grossberg, 2000) and create consistent 3D boundary and surface percepts. Note the binocular interaction in layer 3B of spatially displaced inputs from monocular left and right eye simple cells with the same polarity in layer 4, before opposite polarity binocular simple cells input to complex cells in layer 2/3A [reprinted with permission from Cao and Grossberg (2005)].

The above discussion noted that complex cells in V1 receive binocular inputs, but not how this occurs within laminar cortical circuits in a way that can support binocular vision, including boundary grouping in depth. The 3D LAMINART model proposes and simulates key properties of the anatomical, neurophysiological, and perceptual properties of the brain networks that support vision, including how complex cells in layer 2/3 of V1 become binocular, indeed disparity selective, as well as of how simple, complex, hypercomplex, and bipole cells in cortical areas V1, V2, and beyond support binocular vision (Figure 7; Grossberg and Howe, 2003; Grossberg, 2003; Grossberg and Swaminathan, 2004; Yazdanbakhsh and Grossberg, 2004; Cao and Grossberg, 2005, 2012; Grossberg and Yazdanbakhsh, 2005; Berzhanskaya et al., 2007; Bhatt et al., 2007; Grossberg et al., 2008; Léveillé et al., 2010). The present overview will just summarize how the model proposes that complex cells develop to represent disparity-sensitive properties within the laminar cortical circuits in V1.

### Disparity-Selective Complex Cells in Layer 2/3 of V1

As noted above, complex cells pool inputs from opposite polarity simple cells with similarly oriented receptive fields. Complex cells can hereby respond all along an object’s boundary even if its contrast polarity with respect to the background reverses as the boundary is traversed. Layer 2/3 is known to implement this kind of contrast-invariant boundary detection (e.g., Hubel and Wiesel, 1962; Poggio, 1972; Katz et al., 1989; Alonso and Martinez, 1998). It is also known that monocular, polarity-selective simple cells exist in layer 4 (Hubel and Wiesel, 1962, 1968; Schiller et al., 1976; Callaway, 1998). These properties raise the question: How do the monocular, polarity-selective simple cells in layer 4 get transformed into binocular, disparity-selective, contrast-invariant complex cells in layer 2/3?

Grossberg and Howe (2003) proposed that this occurs in two stages (Figure 7): first, pairs of same-polarity, like-oriented, *monocular* simple cells, that respond to opposite eyes at nearby positions in layer 4, input to same-polarity, like-oriented, *binocular* simple cells at an intermediate position in layer 3B. These binocular simple cells respond selectively to a narrow range of binocular disparities. This processing stage clarifies how inputs from the two eyes binocularly fuse cells that are sensitive to the same polarity, but not to opposite contrast polarities (Julesz, 1971; Poggio and Poggio, 1984; Read et al., 2002). This *same sign* property is one of several that help to guarantee that only monocular cell responses from the left and right eyes that arise from the same object can be binocularly fused. These binocular simple cells can develop from their monocular simple cell inputs using properties of SOM that were summarized above.

Second, pairs of *opposite-polarity*, like-oriented, binocular simple cells at the same position in layer 3B learn to input to *contrast-invariant*, like-oriented, binocular complex cells in layer 2/3 using the above adaptive filtering and rebound properties. These hypotheses are supported by facts such as: layer 4 cells output to layer 3B, but not to layer 2/3 (Callaway, 1998); layer 3B projects heavily to layer 2/3 (Callaway, 1998); and layer 2/3 contains a large number of binocular and complex cells (Poggio, 1972). These complex cells can develop using the properties of opponent rebounds that were summarized above.

This summary does not go into multiple subtleties that are explained in the archival modeling articles, such as the slight differences in the orientation preferences of monocular simple cells of opposite eyes that are activated by viewing an object boundary in-depth, and that is fused at binocular simple cells.

### Alternative Models of V1 Complex Cells

Various alternative models of complex cells have been proposed. For example, Tao et al. (2004) have suggested a model of the neuronal dynamics in the input layer 4Cα of LGN output signals to cortical area V1. They propose to explain how both simple and complex cell responses are found in this layer, and that “through a balance of strong recurrent excitation and inhibition this model yields complex responses in those cells with relatively little LGN drive.” Chance et al. (1999) have proposed that phase-specific outputs of excitatory simple cells drive cells that are coupled together in an excitatory recurrent network. In particular, these authors propose that “local recurrent connections…are responsible for the spatial-phase invariance of complex-cell responses…neurons exhibit simple-cell responses when recurrent connections are weak and complex-cell responses when they are strong, suggesting that simple and complex cells are the low- and high-gain limits of the same basic cortical circuit” (p. 277).

These models are underconstrained in the sense that they do not explain how critical properties, such as the binocular disparity-selective responding of complex cells is realized. They also do not explain how monocular simple cells and binocular complex cells are proposed to support 3D figure-ground separation and both 3D boundary and surface perception. These accomplishments of the visual cortex have been modeled as part of the 3D LAMINART model, whose model cell types and interlaminar interactions have been supported by multiple anatomical, neurophysiological, and psychophysical experiments (e.g., Grossberg and Raizada, 2000; Raizada and Grossberg, 2001, 2003; Grossberg and Swaminathan, 2004; Cao and Grossberg, 2005, 2012, 2014, 2018; Fang and Grossberg, 2009; Grossberg, 2016a). These articles also provide comparative discussions of other models of visual cortex that do not attempt to explain such data; e.g., Raizada and Grossberg (2003, Section 7).

As noted above, one motivation for these models is the fact that both simple and complex cell properties can be recorded in cortical layer 4. There are several possible reasons for this fact, due to both bottom-up and top-down influences, all of them consistent with the theoretical perspective taken in this article. A bottom-up explanation would note that, just as there may be varying degrees of ocular dominance across cells in the V1 cortical map (LeVay et al., 1978; Kara and Boyd, 2009), if only due to the statistical nature of map development, so too may there be gradients of polarity-selective vs. polarity-pooling bottom-up interactions there. A top-down explanation would note that complex cells in layer 2/3 of V1 feed back to simple cells in layer 4 of V1, thereby mixing, albeit with a brief temporal delay, their polarity-invariant properties with the polarity-specific properties of simple cells. Sorting out these various possibilities would benefit from more detailed statistical analyses of both experiments and models of V1 cortical development.

## Homologs of Ocular Dominance Columns in Other Developing Cortical Modalities: Strip Maps

The preceding sections focused on models of visual cortical development and architecture because psychological and neurobiological studies of vision were some of the earliest ones made and because they enjoy one of the largest interdisciplinary databases in science. Other neural models of cortical development have shown that several modalities use variants of the same design principles and mechanisms that support visual cortical development. The following text reviews and unifies some highlights of their properties.

### Strip Maps in Multiple Modalities

A key property is that all the maps exploit variations of how a single ocular dominance column can be used to represent multiple orientations of images that excite an eye at a given position. In all the other examples that will now be summarized, a strip of cells represents a given property that is also used to represent an ordered series of changes in another property. Such a design is accordingly called a *strip map*. A strip map provides enough cortical representational space for the ordered values of the second property to be represented in a map that also codes the first property. Examples include how cortical maps develop to represent the following kinds of information: place-value numbers, auditory streams, speaker-normalized speech, and cognitive working memories that can store repeated items; e.g., ABACBD. These maps occur in both the ventral What cortical stream and the dorsal Where cortical stream, and at multiple levels of the cortical hierarchy.

### Development of Place-Value Numbers and Numerical Comparisons

**Figure 8:**
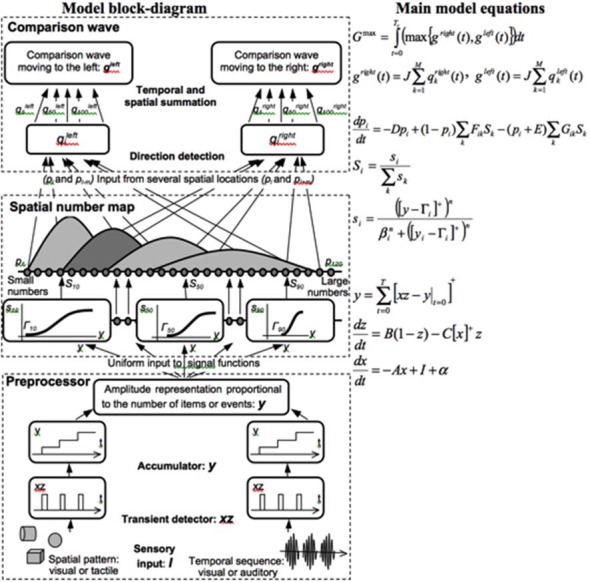
Processing stages of the SpaN model. Preprocessor: For each sensory input increments the activity of the integrator *y*. Integrator activity uniformly activates the spatial number map. Spatial number map: Each activity *p_i_* receives the output *S_i_* that is activated by the integrator input *y*. The signal functions *s_i_* that give the rise to *S_i_* has increasing thresholds and slopes at each successive map cell *i*. Examples for cells 10, 50, and 100 are shown on the diagram. Each pattern *p_i_* “bump” on the spatial number map represents the analog numerical map value of increasing numbers of inputs in a sequence. Comparison wave: see the archival article for details [reprinted with permission from Grossberg and Repin (2003)].

Both animals and humans are capable of representing and comparing numerical quantities. This competence is supported by a spatial map in the inferior parietal cortex that represents small numbers in order of increasing size (Dehaene et al., 1996; Pinel et al., 1999; Piazza et al., 2004). Rhesus monkeys are also known to represent the numerosities 1–9 on an ordinal scale (Brannon and Terrace, 1998, 2000). Only humans, however, have evolved multi-digit place-value number systems whereby much larger numbers can be represented by such a map. Grossberg and Repin (2003) proposed a neural model that is called the Spatial Number Network, or SpaN model (Figure 8), to explain and simulate how small numbers are represented in an ordered spatial map in the inferior parietal cortex of the Where cortical processing stream. Multi-digit place-value numerical representations are proposed to develop through learned associations between categorical language representations in the What cortical processing stream and the Where spatial representation. For example, learned language categories that symbolize separate digits, such as “one,” “two,” “three,” etc., as well as place markers like “ty,” “hundred,” “thousand,” etc., are together associated through learning with the spatial locations of the Where spatial representation. The model that realizes this expanded numerical capability is called the Extended SpaN, or EspaN, model.

**Figure 9:**
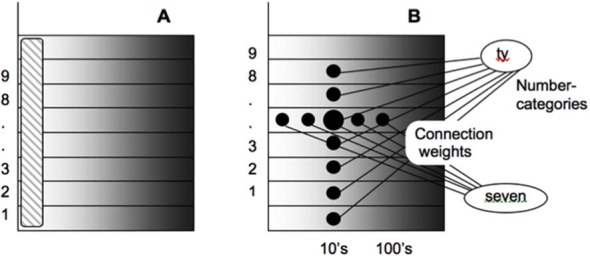
Summary of how place-value numbers are learned *via* What-to-Where interstream information fusion. **(A)** The striped vertical area on the left denotes the primary analog number map in the Where cortical stream. Horizontal strips respond to the same numbers as the corresponding cells in the primary map. **(B)** How What-to-Where associations activate a spatial representation of the number *seven-ty* in the strip that corresponds to *seven* in the primary number map. Sizes of the solid circles encode activities of cells in the strip map. Convergent associations from language representations of “seven” and “ty” maximally activate the cells representing “seventy” [reprinted with permission from Grossberg and Repin (2003)].

As noted in Figure 9A, each numerical representation in the primary analog number map is expanded into a strip map that provides enough representational space for the learning of place-value numbers. Such a strip is a kind of *numerical hypercolumn*. A number that activates a given analog numerical representation in the primary number map can also activate the entire strip corresponding to that numerical representation. For example, the number “seven” would send inputs to its entire strip (Figure 9B). Different place values, such as “ty,” “hundred,” “thousand,” and so on, initially send broadly distributed adaptive signals to the entire strip map. After unsupervised learning, they can activate different positions within each strip, with numbers like “seven,” “seventy,” and “seven hundred” being represented in progressively more distant positions from the primary number map. This ordering emerges from how the learning spontaneously develops when the entire strip map also includes competitive interactions both within and across strips. Grossberg and Repin (2003) hereby demonstrated how a place-value number system develops as an emergent property of What-to-Where interstream information fusion. Piazza et al. (2007) have provided additional experimental fMRI support of this conception by reporting “a magnitude code common to numerosities and number symbols in human intraparietal cortex.”

This summary will not detail the large body of psychophysical and neurobiological data that these models have explained. In particular, the model quantitatively simulates error rates in quantification and numerical comparison tasks, and reaction times for number priming and numerical assessment and comparison tasks. The dynamics of numerical comparison are encoded in activity pattern changes within this spatial map that cause a “directional comparison wave” with properties that mimic data about numerical comparisons, such as the Number Size Effect and the Number Distance Effect (Dehaene, 1997). To clarify how these mechanisms may have arisen through evolution—that is, to explain “where numbers come from”—Grossberg and Repin (2003) noted that these mechanisms are specializations of neural mechanisms that had earlier been used to model data about motion perception, attention shifts, and target tracking, an explanation which clarifies how numerical representations may have evolved from more primitive functions that are known to occur in the cortical Where processing stream, and how inputs to the parietal numerical representation arise.

### Development of Auditory Streams and the Cocktail Party Problem

Auditory communication often takes place in an environment with multiple sound sources simultaneously active, as when we talk to a friend at a crowded noisy party. Despite this fact, we can often track our friend’s conversation, even though harmonics from other speakers’ voices may overlap those of our friend’s voice. Such a conversation is possible because the auditory system can often separate multiple overlapping sound sources into distinct mental objects, or auditory streams. This process has been called *auditory scene analysis* (Bregman, 1990) and enables our brains to solve what is called the *cocktail party problem*.

Many models of auditory scene analysis have been proposed since Bregman’s seminal book. Some of them apply classical engineering methods, such as Independent Component Analysis, to separate independent sources of activity from recorded mixtures of acoustic sources; e.g., Brown et al. (2001). This particular method works if the sources are non-Gaussian signals that are statistically independent of each other. If N sources are present, then at least N observations (e.g., microphones) are needed to recover them^1^. Models of auditory scene analysis vary considerably in their biological plausibility and their ability to detect and track auditory streams without external supervision. An issue of particular importance is whether and how source separation occurs when the auditory signals are significantly occluded by noise. See Szabó et al. (2016) for a recent review.

**Figure 10:**
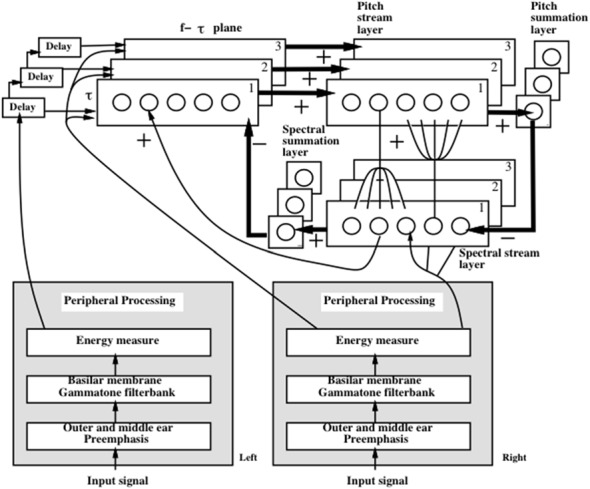
The ARTSTREAM model explains and simulates key data about how auditory scene analysis occurs by combining pitch and source direction information. Multiple auditory streams (labeled 1, 2, 3) can emerge within strip maps that are perpendicular to the primary spectral representation within the spectral stream layer. Spectral-pitch resonances develop between the spectral stream layer and the pitch stream layer to track each acoustic source. These are denoted by the bottom-up and top-down filters with branching pathways between these layers, combined with top-down lateral inhibition *via* the pitch summation layer to realize the ART Matching Rule. Interactions of the ART Matching Rule and asymmetric competition mechanisms in cortical strip maps explain how a source selects the consistent frequencies in its own stream while separating other acoustic signals into another stream. Signals from both ears combine to compute source direction, which also helps to identify a source [adapted with permission from Grossberg et al. (2004)].

Grossberg et al. (2004) introduced the ARTSTREAM neural network model of auditory scene analysis to explain how auditory scene analysis can be achieved by applying basic ART principles and mechanisms in the auditory domain, notably the ART Matching Rule for attentional focusing and resonant conscious awareness, and how it can be used to select and complete multiple auditory streams in noise (Figure 10). To separate multiple auditory streams, the ARTSTREAM model uses variations of the strip maps that represent place-value numbers (Figure 9). These ART and strip map designs together provide a secure biological foundation for ARTSTREAM by suggesting how auditory scene analysis may have emerged during evolution by exploiting widely used brain mechanisms and circuits, while also ensuring that its representations may be incrementally learned in real-time, without experiencing catastrophic forgetting, even in response to noisy and nonstationary acoustical environments.

In the ARTSTREAM model, multiple stages of auditory preprocessing generate a spatial map of tonotopically ordered sound frequencies in log-polar coordinates. This map is often called a *spectral representation*. In order to carry out an auditory scene analysis, a brain uses its spectral representation to learn representations of the pitch of a sound and its spatial location (Bregman, 1990). The front end of the ARTSTREAM model accordingly uses the SPINET, or Spatial PItch NETwork, of Cohen et al. (1995; see gray boxes in Figure 10) to model how the pitch representations arise and simulate many data about pitch perception to support its proposed neural mechanisms.

Using its spectral, pitch, and location representations, ARTSTREAM can track several auditory streams simultaneously. These several requirements build upon a strip map that extends the spectral representation to enable multiple streams to be represented, just as the EspaN model enables place-value numbers to be represented. In the ARTSTREAM strip map, when a sound frequency activates its primary spectral representation, it also activates the entire strip of cells that encode that sound frequency, in much the same way that ordered representations of small numbers in the primary number map expand their representations into strips that can represent place-value numbers. In Figure 10, the anatomical substrate of each stream in the *spectral stream layer* is denoted by a different integer (1, 2, 3). Each stream includes a complete copy of the spectral representation. The strips are perpendicular to the streams, and redundantly represent each different frequency in all the streams.

A stream in the model forms as a result of a *spectral-pitch resonance* that emerges during feedback interactions between the spectral representation of a sound source in the strip map and a representation of its pitch in the pitch stream layer (Figure 10). This pitch representation is a learned recognition category, much like any such category in an ART network. The following processing steps illustrate model dynamics:

First, a sound is transformed into a spatial pattern of frequency-specific activations across the spectral stream layer (Figure 10). These frequencies typically activate the harmonics of a sound’s pitch, at least for the sonorant types of sounds that have a pitch, such as vowels, due to the way in which such sounds are processed in the cochlea. Each frequency activates its entire strip. The cells that are activated by these sound frequencies then send bottom-up signals through an adaptive filter. Because the harmonics of the sound’s pitch are active, this filter is sometimes called a *harmonic sieve* (Duifhuis et al., 1982; Scheffers, 1983).

Output signals from this filter activate a subset of cells within the pitch stream layer (Figure 10) before these cells compete to choose the most active cell population. Because such a cell selectively responds to the harmonics of a pitch, it is called a *pitch category*. A chosen pitch category, in turn, activates a top-down expectation that obeys the ART Matching Rule. Because the top-down expectation obeys the ART Matching Rule, spectral components are selected if they match harmonics of the active pitch category, and are suppressed if they do not. In this way, noise is suppressed that would otherwise occlude processing the pitch’s spectral components. Reciprocal bottom-up and top-down excitatory signals then resonate between the spectral and pitch stream layers. Such a resonance provides the temporal coherence that allows one voice or instrument to be tracked through a noisy environment that contains multiple active sound sources.

A proper balance of cooperation and competition is needed to choose multiple streams while suppressing noise. Asymmetric intrastrip competition is stronger from the primary spectral representation to other positions on its strip, than conversely; that is, from stream 1 to streams 2 and 3. Interstrip competition also occurs, and is regulated by active top-down expectations that obey the ART Matching Rule.

The intrastrip competition enables the primary spectral representation (labeled 1 in Figure 10) to win the competition for generating the first spectral-pitch resonance. It hereby becomes the first stream to be active. The harmonic components that resonate with the chosen pitch category can also use the asymmetric intrastrip competition to inhibit these components at other strip positions that are distal to the primary spectral representation (labeled 2 and 3 in Figure 10).

In contrast, the frequencies that are inhibited in the primary stream by pitch-activated top-down signals that obey the ART Matching Rule cannot inhibit themselves at positions distal to the primary spectral representation. These uninhibited frequencies can then activate the bottom-up filter at positions distal to the primary spectral representation (e.g., in stream 2), so that another pitch category can be chosen, and can begin to resonate with a subset of spectral stream cells, thereby creating another spectral-pitch resonance within stream 2. And so on, thereby enabling several spectral-pitch resonances to simultaneously represent several auditory streams. These specialized versions of Adaptive Resonance Theory, or ART, mechanisms also clarify how spatial location cues can help to disambiguate two sound sources with similar spectral cues (see the f – τ plane in Figure 10).

### Auditory Continuity Illusion, Separation of Intersecting Frequency Sweeps, and Scale Illusion

The ARTSTREAM model simulates data from streaming experiments, such as how the *auditory continuity illusion* occurs, during which a tone is perceived to continue through a noise burst even if the tone is not present during the noise; how a tone sweeping upwards in frequency creates a bounce percept by grouping with a downward sweeping tone due to proximity in frequency, even if noise replaces the tones at their intersection point; and how the scale illusion of Deutsch occurs (Deutsch, 1975), whereby downward and upward scales presented alternately to the two ears are regrouped based on frequency proximity, leading to a bounce percept.

ARTSTREAM would need to be further developed to explain more complex streaming data. ARTSTREAM models only a single scale of sustained detectors, or detectors of a given size that respond to sounds with sustained energy across time in particular frequencies. Grossberg et al. (2004) noted that preprocessing by detectors of multiple scales that are sensitive to transient sounds are needed, in addition to sustained detectors, to fully explain data about how frequency sweeps are separated, as well as other acoustic signals with rapidly changing frequencies. Parallel interacting streams of sustained and transient cells are also used to explain data about how speech and language sounds are represented at higher levels of brain processing (e.g., Cohen and Grossberg, 1997; Boardman et al., 1999). The circuit properties that give rise to these transient cells can also be used to guide the design of transient cells at earlier processing stages.

For present purposes, the main point is that multiple auditory streams and their spectral-pitch resonances build upon strip maps, abetted by intrastrip and interstrip competitive interactions, just as in the case of place-value numbers.

### Development of Speaker Normalization and Language by Circular Reactions

Different speakers—such as young children, women, and men—utter language utterances in different frequency ranges. Despite this variability, we can all understand each other’s language utterances without having to separately learn language meanings for each different frequency range that any speaker might use. The transformation of individual speaker frequency ranges into a format that can be understood in a speaker-invariant way is called *speaker normalization*. Various engineering approaches to speaker normalization have been proposed; e.g., Joy et al. (2018). The biological neural model that is proposed below again uses ART, strip maps, and asymmetric competitive interactions to achieve speaker normalization, and is moreover homologous to the model used for auditory streaming.

Speaker normalization is essential for babies to learn language from adult speakers, since their parents’ spoken frequencies differ from those that the baby can *babble*. Babies babble simple sounds during a critical period of their development. They also hear their own babbled sounds and learn to associate the heard sounds with the motor commands that they used to make them. This feedback loop between speaking and hearing is called a circular reaction (Piaget, 1963).

**Figure 11:**
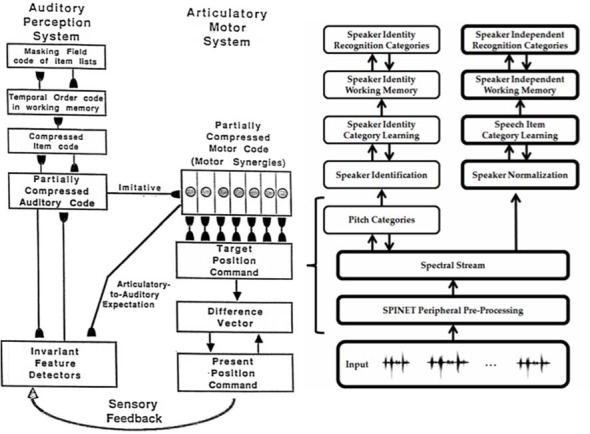
(left architecture) An auditory-to-articulatory feedback loop enables babbled sounds to activate learning in an imitative map that is later used to learn to reproduce the sounds of other speakers. The invariant feature detectors include preprocessing by speaker normalization circuits. An articulatory-to-auditory expectation renders learning possible by making the auditory and motor data dimensionally consistent [reproduced with permission from Cohen et al. (1988)]. (right architecture) Parallel streams in the ARTSPEECH model learn speaker-independent speech and language meaning, including a mechanism for speaker normalization (right cortical stream), and for learning speaker-dependent vocalic qualities (left cortical stream) [reproduced with permission from Ames and Grossberg (2008)].

How do babies use these relatively simple learned associations to learn the more complex language utterances of adult speakers? By converting all speech sounds into a frequency-normalized format, including the sounds that the baby babbled, speaker normalization enables sounds from adult caretakers to be filtered by the speaker-normalized map that was learned during babbling, and to thereby enable the baby to begin to imitate heard sounds as part of its own language productions. This kind of map is accordingly called an *imitative map* (Figure 11, left panel).

The NormNet model of Ames and Grossberg (2008) simulates speaker normalization to generate a pitch-independent representation of speech sounds, while also preserving information about speaker identity in a parallel cortical stream (Figure 11, right panel). The model exploits the fact that auditory streams form before the stage where speaker normalization occurs. In this way, our brains can normalize the frequencies of a single attended voice after it is segregated in a stream.

**Figure 12:**
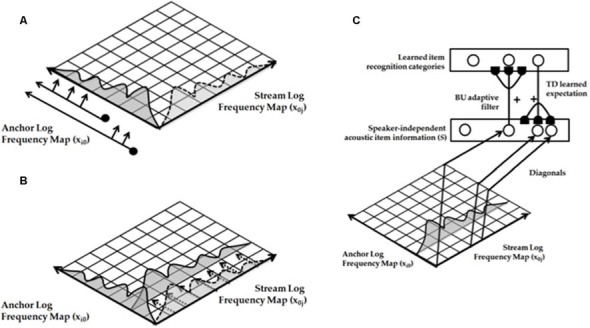
**(A)** Anchor Map and Stream Map: these strip maps are organized so that their strips overlap each other. Both maps receive the spectral representation, in log frequency coordinates, from the streamed sound. **(B)** Coincidence detection: chosen by asymmetric competition, the winning Anchor Frequency Coding Cell triggers coincidence detection along its Anchor Frequency Strip, which also receives spectral inputs from the Stream Map. Activations of the Stream Map are thereby translated into the Anchor Frequency Strip. **(C)** Speaker-independent spectral representation: summation of activities along each diagonal strip creates a speaker-independent spectral representation, which is then fed into an ART network that learns to categorize it [adapted with permission from Ames and Grossberg (2008)].

Remarkably, both auditory streaming and speaker normalization in the model uses multiple strip representations and asymmetric competitive interactions (Figure 12), thereby suggesting that these two circuits, with their strikingly different functions, arose from similar neural designs.

In the auditory cortex of both humans and other mammals, multiple tonotopic maps exist that contain strips of cells called iso-frequency contours that respond to a specific best frequency (Merzenich and Brugge, 1973; Imig et al., 1977; Morel and Kaas, 1992; Morel et al., 1993; Hackett et al., 1998; Kaas and Hackett, 1998, 2000; Formisano et al., 2003; Rauschecker and Tian, 2004; Petkov et al., 2006). NormNet assumes that two identical tonotopic strip maps occur with perpendicular, or at least overlapping, orientations (Figure 12A). These maps are called the Anchor Log Frequency Map (Anchor Map) and the Stream Log Frequency Map (Stream Map). Both the Anchor Map and Stream Map are activated by a speech sound, which is assumed to have already been segregated into an auditory stream by processes such as those modeled by ARTSTREAM.

Due to this spatial format, activations in these strips intersect. Asymmetric competition occurs between frequencies in the Anchor Map to choose the cell with the lowest active frequency in the speech sound, which typically has the greatest spectral energy (Figure 12A). This cell is called the *anchor frequency coding cell*. While the anchor frequency coding cell wins the asymmetric competition, it inhibits activations corresponding to higher frequencies in the Anchor Map. Coincidence detection between the perpendicular strips determines which cells will next be activated. In particular, only cells in the Stream Map that receive acoustic inputs and that intersect the strip of the anchor frequency coding cell will be activated (Figure 12B). In other words, such coincidences occur in the strip corresponding to the Anchor Frequency of the Anchor Map and all the active strips corresponding to spectral activations in the Stream Map.

These coincidences are registered by diagonally connected strips that transform the Anchored Stream into a speaker-normalized representation *S* (Figure 12C). In particular, each cell in the *S* field sums inputs from all cells within diagonal stripes that cross the maps. This speaker-normalized representation then triggers the learning of speaker-normalized recognition categories. In Ames and Grossberg (2008), category learning is carried out by a fuzzy ARTMAP network with default parameters (Carpenter et al., 1992).

NormNet was tested by simulating how its normalized speech items are categorized and stably remembered by ART circuits. The simulated acoustic inputs were synthesized steady-state vowels from the Peterson and Barney (1952) vowel database and achieved accuracy rates similar to those achieved by human listeners.

### Development of Item-Order-Rank Working Memories

Essentially all higher-order biological intelligence requires that *sequences* of previously experienced items or events be used to determine subsequent thoughts, decisions, and actions. Working memory temporarily stores such sequences until they are performed or used to trigger learning and LTM of sequence categories, or *list chunks*, with which to control future behaviors. Due to its importance of working memory in controlling intelligent choices and predictions in complex situations, the psychological and neurobiological study and modeling of working memory has been very active for decades; e.g., Miller et al. (1960), Atkinson and Shiffrin (1968), Baddeley and Hitch (1974), Baddeley (2010) and Constantinidis and Klingberg (2016). As with all the kinds of models reviewed in this book, these models vary greatly in their biological plausibility and in their ability to support learning. For example, in the pioneering Atkinson and Shiffrin (1968) model, previously stored items move from one storage slot to the next as a new item is stored. Such a model faces both conceptual and empirical problems. For example, it cannot explain the ubiquitous bowed curves that are found in working memory data (see below) and it cannot learn list chunks.

Multiple working memories are used to temporarily store sequences of linguistic, spatial, and motor items, among others, such as sequences of words, navigational goals, and arm movement commands. It is explained below why all working memories are realized by a similar kind of recurrent neural network, despite their different functions. Another important property of working memories is that they can store repeated items, such as the list ABACBD. The model of working memory proposed below uses strip maps to realize this property, as well as ART to categorize sequences of stored items.

A series of articles, starting in 1978, have characterized how biological working memories are designed, and have explained and predicted many psychological and neurobiological data about them (e.g., Grossberg, 1978a; Grossberg, 2018; Grossberg et al., 1997; Boardman et al., 1999; Grossberg and Myers, 2000; Grossberg and Pearson, 2008; Silver et al., 2012; Kazerounian and Grossberg, 2014; Grossberg and Kazerounian, 2016). These articles also explained why and how all working memories, whether linguistic, spatial, or motor, all share the same underlying kind of neural circuit; namely, a specialized recurrent on-center off-surround network whose cells obey membrane, or shunting, equations.

These working memories have been derived from postulates which ensure that list chunks can be learned and stably remembered. Working memories that obey the *LTM Invariance Principle* have this property. The LTM Invariance Principle guarantees, for example, that the first time a novel word, such as MYSELF, is stored in working memory, it does not force catastrophic forgetting of previously learned list chunks that code for its familiar subwords MY, ELF, and SELF. Without such a property, language, spatial, and motor sequential skills could not be learned.

It was shown, starting in Grossberg (1978a), how the LTM Invariance Principle could be satisfied by a working memory for which temporal sequences of items or events are converted into an evolving spatial pattern of activity over item chunks that store these events in working memory. Then, during rehearsal, the item stored with the largest activity is rehearsed first, the item with the next largest activity is rehearsed second, and so on. In other words, a *spatial gradient* of activity across item chunks encodes both the items that are stored in working memory and their temporal order. Such working memories are thus called Item-and-Order working memories. The LTM Invariance Principle is satisfied if the relative sizes, or ratios, of activities in this spatial gradient remain the same as new items are stored in working memory, even if their total sizes may change through time to approximately normalize the total activity that is stored by the recurrent shunting network.

If a *primacy gradient* is stored, then the first item is stored with the largest activity, the second item is stored with the next largest activity, and so on. Rehearsal from a primacy gradient can recall the items in the correct order in which they were stored. If a *recency gradient* is stored, with the last item having the largest activity, then items are rehearsed in the reverse order that they were stored, with the last item recalled first. If a *bowed gradient* is stored, with items at the beginning and end of the list stored with larger activities then items in the middle, then items at the beginning and end of the list are rehearsed before items in the list middle. Remarkably, as more items get stored, a primacy gradient is always converted into a bowed gradient. As a result, sufficiently long lists cannot be recalled in their correct temporal order from working memory.

A series of articles has illustrated the explanatory power of the hypothesis that all linguistic, spatial, and motor working memories use variations of a recurrent shunting on-center off-surround network. Data about bowed serial position effects are ubiquitous in the working memory literature, notably psychological data about the *linguistic* working memories whereby humans do immediate serial recall, and immediate, delayed, and continuous distractor free recall; and neurophysiological data recorded from monkeys about the *motor* working memories whereby planned arm movement sequences are stored and performed. Grossberg and Pearson (2008) and Grossberg (2017b) explain and quantitatively simulate these and other psychological and neurobiological data about working memory using homologous Item-Order-Rank working memories. Silver et al. (2012) use homologous Item-Order-Rank working memories to explain and simulate the *spatial* working memories whereby movement storage, planning, and control of sequential saccadic eye movements is achieved.

Working memories also need to be able to store sequences of events that may repeat themselves; e.g., ABACBD. This generalization of Item-and-Order working memories is called an Item-Order-Rank working memory. Item-Order-Rank working memories provide a foundation for learning both speech and language. It has, for example, been shown that a suitably designed three-level network of Item-Order-Rank working memories can store and learn sequences of repeated words, such as “DOG EATS DOG.”

Strip maps enter the story by enabling Item-Order-Rank working memories to store item sequences that contain repeats. Each of these strips is a kind of *item-rank hypercolumn*. For example, to store a list like ABACBD, the item representation of A would activate its item-rank hypercolumn in rank positions 1 and 3, B would activate its item-rank hypercolumn in rank positions 2 and 5, C in rank position 4, and D in rank position 6. The rank information is proposed to be projected to the prefrontal cortical working memory from numerical representations in the parietal cortex. This prediction uses properties of the Spatial Number Network, or SpaN, model of Grossberg and Repin (2003), that was described above, of how numerical maps in the inferior parietal cortex enable numerical quantities to be represented and compared. Properties of SpaN model neurons were supported by neurophysiological data of Nieder and Miller (2003, 2004), who also reported prefrontal projections of parietal numerical representations. In such an Item-Order-Rank working memory, relative activity still represents the temporal order of a sequence that is stored, and the off-surround of the network can still equally inhibit all other cells, including the cells in each item-rank hypercolumn.

Item-Order-Rank working memories have been used as part of larger neural architectures for learning and performing sequential tasks. One such architecture is the lisTELOS architecture of Silver et al. (2012) which explains and simulates how an Item-Order-Rank working memory in the prefrontal cortex stores and learns multiple spatial positions with which to control sequences of eye movements. Model simulations reproduced behavioral, anatomical, and electrophysiological data from multiple experimental paradigms, including visually-guided and memory-guided single and sequential saccadic eye movement tasks, and behavioral data from two microstimulation paradigms in which the supplementary eye fields were stimulated, thereby explaining how their seemingly inconsistent findings about saccade latency could be reconciled. It is explained below why similar working memory circuits and architectures can store and learn multiple types of sequential behaviors.

## Topographic Maps of Feature Detectors and Their Gaussian Peak Shifts

Not all feature-selective maps need strip maps in order to function well. Several examples of this will now be summarized in order to illustrate the diversity of possibilities.

### Steering During Optic Flow Navigation

Visually guided navigation enables humans and many other animals to move through cluttered natural scenes without colliding with obstacles. At least two parallel processes, with computationally complementary properties (Grossberg, 2000), contribute to this competence.

The first process uses the *optic flow* that is generated as an animal moves with respect to its environment in order to steer towards a goal. Optic flow is the information carried by the light that streams in time over the retina due to such movements (Gibson, 1950). For example, if the movement is straight ahead through a rigid environment, then the optic flow generates a radial motion pattern whose individual motion vectors emanate from a single position, which is called the focus of expansion. *Heading* is the direction that the observer is traveling at any time, and can be computed from a combination of optic flow information and outflow movement commands, called corollary discharges or efference copies, that code eye and head movements relative to the body. Browning et al. (2009a) provide a comparative review of the three main classes of heading models: differential motion, decomposition, and template models.

The second process separates objects—including goal objects and obstacles—from each other and the background using their relative motion to enable tracking of a goal without bumping into obstacles. Browning et al. (2009b) and Elder et al. (2009) review alternative models and data about tracking.

These two processes are computationally complementary because optic flow computations use *additive* processing of motion signals across an entire scene to compute an observer’s heading, whereas *subtractive* processing separates an object’s boundaries as they move relative to a background. These processes occur due to interactions between cortical areas MT^−^ and MSTv to compute heading, and between cortical areas MT^+^ and MSTd to control tracking. Supportive neurophysiological data for these hypotheses is provided in Born and Tootell (1992).

The ViSTARS (Visually-guided Steering, Tracking, Avoidance, and Route Selection) neural model (Browning et al., 2009a,b; Elder et al., 2009) proposes how primates use these two kinds of complementary motion information to segment objects and determine heading for purposes of goal approach and obstacle avoidance. The model simulated this competence in response to video inputs from real and virtual environments and thereby produced trajectories that match those of human navigators.

**Figure 13:**
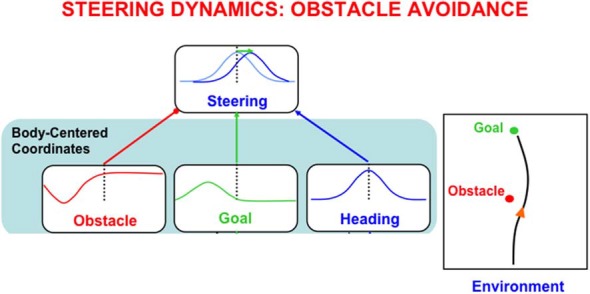
A peak shift due to the negative Gaussian of an obstacle prevents a collision with the obstacle without losing the information that is coded in the sum of the goal and heading Gaussians of how to reach the goal [adapted with permission from Browning et al. (2009b)].

A topographic map enters the story because it is known that, during human visually-based navigation, goals behave like *attractors* and obstacles behave like *repellers* (Fajen and Warren, 2003). The ViSTARS model shows how a topographic map of movement directions can realize this kind of attractor-repeller control, leading to navigational trajectories that closely match human performance. In this map, the instantaneous directions of heading, goal object, and obstacle are represented by Gaussian activity profiles (Figure 13). The heading and goal object Gaussians add whereas the obstacle Gaussian activity profile is subtracted from them, thereby causing a *peak shift* of the direction of heading away from the obstacle.

Gaussian receptive fields occur in many topographic cortical maps. Whenever one of them is subtracted from another one whose activation represents a sufficiently nearby position in the feature map, a peak shift occurs in the direction opposite to the peak in the inhibitory Gaussian.

### Peak Shift During Reinforcement Learning: Do We Know What We Like?

A peak shift can, for example, occur in situations where pigeons are trained using operant conditioning to respond to one colored light when it flashes on, and to *not* respond to other colored lights when they flash on. During learning by operant conditioning, animals or humans are rewarded or punished only after they emit a certain behavior, or set of behaviors (Skinner, 1938).

Suppose that a pigeon is trained to respond to a given colored light cue. After learning occurs, the pigeon also responds to other colors *via* a *generalization gradient*; that is, the pigeon responds progressively less as a function of how different the wavelength of the test light is compared to the training light. This result implies that color representations are organized in a topographic map. Further corroboration of this hypothesis can be found by additional training of the pigeon: After training with reward to respond to one colored light, train it with punishment to *not* respond to a different colored light, and do so using *errorless training* (Terrace, 1963). The rewarded wavelength activates an excitatory Gaussian, whereas the punished wavelength activates a negative Gaussian, due to interactions of their cortical sensory representations with affective centers like the amygdala. When pigeon responses are now tested to other colored lights, a remarkable effect called *peak shift and behavioral contrast* is observed.

*Peak shift* means that, just as in the case of optic flow navigation, the pigeon now pecks in response to a color that it has never experienced. This color is “repelled” from the wavelength on which the pigeon was earlier punished. *Behavioral contrast* means that the pigeon responds more to this novel color than it did to the rewarded color!

Grossberg (1975a) proposed that this happens because the net gradient that causes the peak shift is narrower than a single Gaussian gradient. Gaussian gradients are caused in this feature map by on-center off-surround intercellular interactions whose on-center and off-surround have Gaussian receptive fields. These interactions obey shunting laws, so their total activity tends to be approximately normalized. Because fewer active cells cause the net gradient, its peak activity is higher than that of a stand-alone gradient.

When one considers the great variety of ordered feature maps in our brains, it becomes clear that, after a lifetime of rewards and punishments, we may intensely like options that we never before experienced. In this sense, “we may not know what we like.”

### Peak Shifts During Motion Perception of an Object and Its Parts

Optic flow navigation and operant conditioning are not the only situations in which topographic feature maps exist and lead to peak shifts. One particularly interesting one, which will not be further explained here, concerns how we see an object’s parts move relative to the object as the object itself moves in its environment. For example, how do we see a person’s arms swing back and forth with respect to her body as she walks down the street? Such a percept is said to obey a rule of *vector decomposition* (Johansson, 1974).

Grossberg et al. (2011) have explained this percept by simulating how the motion direction of the object, again represented by a Gaussian receptive field, is subtracted from the direction of the object part’s motion direction, which is also represented by a Gaussian, thereby causing a peak shift in the perceived direction of the part’s motion relative to the object. Then objects and their parts are seen moving relative to a common reference frame.

### List Chunks of Variable Length Lists: Masking Fields and Self-similar Map Development

The final example concerns how lists of variable length that are stored in working memory can learn to be categorized during behaviors in real-time (Kazerounian and Grossberg, 2014). The LTM Invariance Principle was described above to show how brains prevent storage in working memory of a novel word, such as MYSELF, from causing catastrophic forgetting of previously learned list chunks that code for its familiar subwords MY, ELF, and SELF. A related problem is: Why is not the brain forced to process the new word as a sequence of its smaller familiar words? How does a not-yet-established word representation overcome the salience of already well-established phoneme, syllable, or word representations to enable learning of the novel word to occur? This is called the Temporal Chunking Problem.

**Figure 14:**
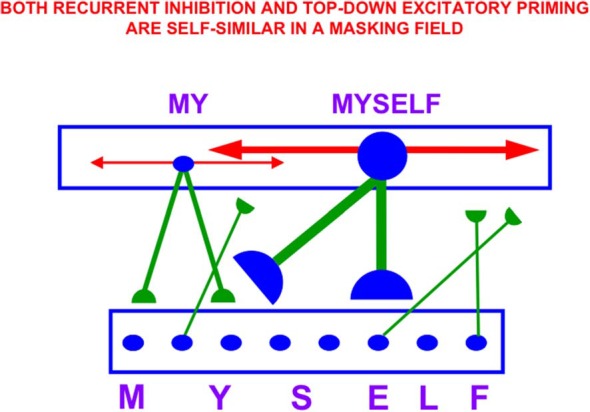
A Masking Field is a recurrent shunting on-center off-surround network with multiple self-similar receptive field sizes. Self-similar growth during development leads to Masking Field list chunks such that longer words activate list chunks with stronger recurrent inhibitory connections and top-down adaptive excitatory connections. As a result, longer lists can preferentially activate larger list chunks (e.g., of MYSELF), which can thereby inhibit chunks of smaller subwords (e.g., MY) more than conversely.

Cohen and Grossberg (1986, 1987) first showed how this problem can be overcome by a Masking Field network by using cells with multiple receptive field sizes, or spatial scales (Figure 14). A Masking Field is a recurrent on-center off-surround network whose cells obey the membrane equations of neurophysiology (shunting laws) that occur, as noted above, with multiple receptive field sizes, or spatial scales. These spatial scales are related to each other by a property of *self-similarity*; each scale’s properties, including its cell body sizes and their excitatory and inhibitory connection lengths and interaction strengths, are (approximately) a multiple of the corresponding properties in another scale. This self-similarity property can develop as a result of simple activity-dependent growth laws in the following way.

During this developmental process, just like in many map development processes, two cortical levels interact. The cells in the cortical level that will eventually represent item chunks are endogenously active during a critical period of development. During this active phase, these cells send growing connections to the cortical level that will eventually represent the Masking Field. Suppose, for simplicity, that these growing connections are distributed randomly across the Masking Field. As a result of this growth, different Masking Field cells will receive different numbers of connections. Due to the endogenous activity, Masking Field cells that receive more connections will, on average, receive a larger total input activity through time.

Activating Masking Field cells above a fixed threshold causes their cell bodies and connections to grow approximately proportionally. This is called *self-similar growth*. Cell growth terminates when the cell bodies become large enough to dilute their activities sufficiently in response to their inputs so that the total inputs no longer exceed the growth-triggering threshold. Because cells that receive more input connections also receive larger total inputs through time, they will grow larger than cells that receive fewer input connections. The effects of individual inputs are thus smaller on the firing of larger cells. Self-similar growth hereby *normalizes* the total effect of all the inputs that converge on a Masking Field cell. Consequently, a Masking Field cell will only fire vigorously if it receives active inputs from all of its item chunk cells. In other words, self-similar growth ensures that each Masking Field cell responds *selectively* to the input sequence that it codes.

Due to self-similar growth, larger Masking Field list chunks selectively represent longer lists because they need more inputs, and thus more evidence, to fire. Once they fire, their stronger inhibitory interaction strengths than those of smaller list chunks can inhibit the smaller list chunks more than conversely. This is another example of asymmetric competition. The stronger inhibition from list chunks of longer, but unfamiliar, lists (e.g., MYSELF) enables them to inhibit the chunks that represent shorter, but familiar, sublists (e.g., MY), more than conversely, thereby solving the Temporal Chunking Problem. Once developed, Masking Fields explains many data about recognition learning, including such phenomena as the Magical Number Seven Plus and Minus Two, the word length effect, and the word superiority effect.

## Rate Gradients Induce Maps for Learning Timing and Spatial Navigation

### How Cell Populations Whose Cells Respond at Different Rates Represent Large Times and Spaces

How do our brains learn to bridge large time intervals of hundreds of milliseconds or even several seconds, and thereby learn to associate events that are separated by time intervals that are behaviorally meaningful? How do our brains learn to represent large spatial regions, such as the large rooms and open fields within which humans and animals can successfully navigate? Answers to both of these questions must confront the fact that the typical biophysics and anatomy of individual neurons do not enable them to span such long times or large spaces. Remarkably, as will be summarized below, spatially ordered populations of neurons that respond at increasingly slow rates can achieve both capabilities. The emergent properties that these populations generate can, moreover, explain challenging parametric psychological and neurobiological data about temporal and spatial properties of behavior.

### Spectral Timing and Spacing in Lateral and Medial Entorhinal-Hippocampal Systems

In particular, large spatial representations emerge within the entorhinal and hippocampal cortices using interactions between entorhinal grid cells and hippocampal place cells. There has been intense experimental and theoretical interest in how these properties emerge since the classical article of O’Keefe and Dostrovsky (1971) reported the existence of hippocampal place cells. John O’Keefe won the Nobel Prize in Physiology or Medicine in 2014 for this and subsequent work on how spatial navigation works. May-Britt and Edvard Moser shared the 2014 Nobel Prize in Physiology or Medicine with John O’Keefe for their discovery of entorhinal grid cells (Hafting et al., 2005).

Grossberg and Pilly (2012) provide a review and comparative analysis of the three main types of model mechanisms that have been proposed for how entorhinal grid cells form: oscillatory interference, 2D attractor, and SOM. Modeling hippocampal place cells has been no less active. Some approaches assume Gaussian receptive fields of place cells that are centered at different positions in a space defined by two-dimensional Cartesian (x, y) coordinates; e.g., Tsodyks and Sejnowski (1995). Such models can then be used to draw conclusions about spatial navigation that are based upon how these place cells may interact in this representation of space. Other approaches combine associative learning and reinforcement learning to generate place cells; e.g., Arleo and Gerstner (2000), but without an influence from grid cells.

My own work with Praveen Pilly attempts to provide a unified theory of the learning processes that support adaptively timed and spatial navigation behaviors (Grossberg and Pilly, 2012, 2014; Pilly and Grossberg, 2012, 2013a,b, 2014). The spatial navigation theory, whose properties are unified in our GridPlaceMap model, has many parsimonious and elegant properties. In particular, grid cells and place cells, despite their dramatically different receptive field properties, may arise during development through a learning process that uses the *same* SOM laws and circuits for learning both types of cells. Both grid cells and place cells are spatial categories in this hierarchy of SOMs. Moreover, each SOM amplifies and learns to categorize the most frequent and energetic co-occurrences of its inputs, while suppressing the representations of less frequent and energetic input patterns using its recurrent inhibitory interactions. The angular and linear velocity signals that drive these learning processes arise from head direction cells and stripe cells that are both modeled by homologous ring attractors. Additional homologs between spatial navigational learning and adaptively timed learning will be summarized below.

This section will sketch an explanation of why spatial and temporal representations that represent large spaces and times, unlike spatial attention and working memory, are both in a single part of the brain. The reason seems to be that, remarkably, these spatial and temporal representations seem to use variations of a single brain design that is characterized by similar equations. In particular, large time intervals can be bridged using a mechanism of *spectral timing* (Grossberg and Schmajuk, 1989; Grossberg and Merrill, 1992, 1996; Grossberg and Seidman, 2006; Franklin and Grossberg, 2017; Grossberg, 2017b) whereby a “spectrum” of *time cells* (MacDonald et al., 2011) along a dorsoventral gradient in the *lateral* entorhinal cortex, each with different reaction rates, can learn to match the statistical distribution of expected delays in reinforcement over hundreds of milliseconds, or even seconds.

The cells in this dorsoventral gradient respond at progressively slower rates from its dorsal to ventral end, and obey a Weber Law whereby cells that respond later do so with a proportionally larger variance of response times. Although each of the cells in such a spectrum reacts at different times, their population response as a whole can learn to bridge, and adaptively time, much longer time intervals at which events can be associated that are separated in time, as occurs during trace conditioning, delayed non-match to sample, and other conditioning paradigms with delays between offset of a conditioned stimulus and onset of an unconditioned stimulus.

In a similar way, large spaces can be navigated using a mechanism of *spectral spacing* (Grossberg and Pilly, 2012, 2014; Mhatre et al., 2012; Pilly and Grossberg, 2014) whereby a “spectrum” of *grid cells* (Hafting et al., 2005; McNaughton et al., 2006; Sargolini et al., 2006) can be learned along a dorsoventral gradient in the *medial* entorhinal cortex. As in the case of spectral timing, the cells in this spectrum, or gradient, respond at progressively slower rates from its dorsal to ventral end and exhibit spatial Weber Law properties. In this case, the Weber Law properties include larger spatial scales and spacing of grid cells as the dorsoventral gradient is traversed that match anatomical and neurophysiological data (Sargolini et al., 2006; Brun et al., 2008; Stensola et al., 2012). A wide range of additional neurophysiological data about cells in the dorsoventral gradient of medial entorhinal cortex can also be explained by this model, including simple ones like phase precession (Pilly and Grossberg, 2013b) and more subtle ones like the way in which reduction of theta rhythm by medial septum inactivation may covary with impaired entorhinal grid cell responses due to reduced cholinergic transmission (Pilly and Grossberg, 2013a).

Contiguous grid cells of different spatial scales can together, using properties of a SOM, drive the learning of *place cells* in the hippocampal cortex (O’Keefe and Dostrovsky, 1971) that can represent large spaces. As noted above, place cells are spatial categories in this SOM. Critically, the spaces that such a place cell can represent are the *least common multiple* of the spatial scales of the grid cells that input to it (Gorchetchnikov and Grossberg, 2007; Pilly and Grossberg, 2012, 2013b). If the grid cells exhibit centimeter spatial scales, the corresponding place cells can represent spaces that are many meters in size. Place cells that are learned from grid cells can thus support navigational behaviors within the large spaces that are characteristic of terrestrial animals. At an early developmental stage, place cells can be directly learned without grid cells, and can perhaps represent the small spaces that rat pups traverse before they leave their nests (Langston et al., 2010; Wills et al., 2010, 2012; Pilly and Grossberg, 2014; Muessig et al., 2015).

### ART Matching Rule Dynamically Stabilizes Learning in the Entorhinal-Hippocampal System

**Figure 15:**
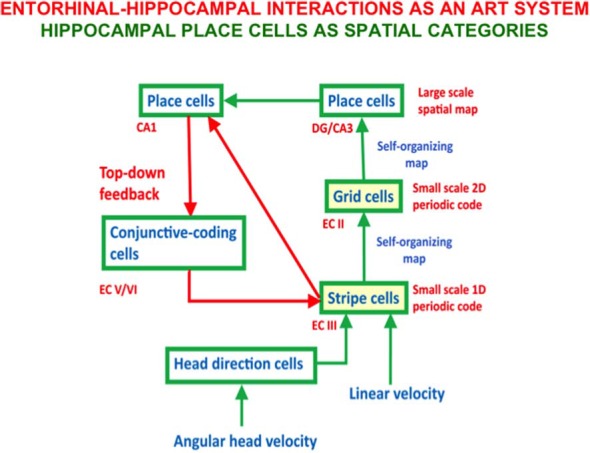
The GridPlaceMap model contains self-organizing maps (SOM) whereby stripe cells learn to activate grid cells, and grid cells learn to activate place cells. In addition, top-down connections from place cells to stripe cells obey the ART Matching Rule and dynamically stabilize both kinds of learning, thereby converting the entire system into an ART model wherein place cells are spatial categories [adapted with permission from Mhatre et al. (2012)].

Place cell selectivity can develop within seconds to minutes and can remain stable for months (Wilson and McNaughton, 1993; Muller, 1996; Frank et al., 2004). This combination of fast learning and stable memory is yet another example of how the brain solves the *stability-plasticity dilemma*. To realize stable memory, the bottom-up SOM interactions that learn grid cells and place cells activate top-down interactions that obey the ART Matching Rule, which dynamically stabilizes the learning of these cells (Figure 15).

Neurophysiological data about the hippocampus from several labs are compatible with ART predictions about the role of top-down expectations and attentional matching in stabilizing learned grid cells and place cells. For example, Kentros et al. (2004) reported that “conditions that maximize place field stability greatly increase orientation to novel cues. This suggests that storage and retrieval of place cells are modulated by a top-down cognitive process resembling attention and that place cells are neural correlates of spatial memory.” In addition, NMDA receptors, which support many learning processes in the brain, also mediate long-lasting hippocampal place field memory in novel environments (Kentros et al., 1998). Compatible data have shown that hippocampal plasticity reflects an “automatic recording of attendee experience” (Morris and Frey, 1997) and that hippocampal inactivation causes grid cells to lose their spatial firing patterns (Bonnevie et al., 2013).

In addition to the fact that both spectral timing and spectral spacing in the entorhinal-hippocampal system seem to exploit a similar temporal rate gradient to develop time cells and grid cells, respectively, spectral timing seems to be a variant of a brain design for adaptively timed learning that is also found in the cerebellum and basal ganglia, where it carries out different behavioral functions (Fiala et al., 1996; Brown et al., 1999; Grossberg, 2016b). In all cases, it seems that the timed spectrum is set up by a calcium gradient that modulates the dynamics of metabotropic glutamate (mGluR) receptors (e.g., Finch and Augustine, 1998; Takechi et al., 1998; Ichise et al., 2000; Miyata et al., 2000). Indeed, all of these neural circuits seem to exploit an ancient design that is found even in non-neural cells, such as HeLa cancer cells, where again a calcium gradient is implicated (Bootman and Berridge, 1996, p. 855). It remains to be seen if the spectral spacing gradient is also induced by a calcium-mediated gradient of mGluR dynamics.

## Concluding Remarks

This article provides a unified overview of several of the design principles and mechanisms that cortical maps use to represent many different types of information across multiple modalities. One design explains how brains use a strip map that simultaneously enables one feature to be represented throughout its extent, as well as an ordered array of another feature at different positions of the strip. Strip maps include circuits to represent ocular dominance and orientation columns, place-value numbers, auditory streams, speaker-normalized speech, and cognitive working memories that can code repeated items. A second design explains how feature detectors for multiple functions develop in topographic maps. Such maps support optic flow navigation, reinforcement learning, motion perception, and category learning at multiple levels of brain organization. A third design explains how a spatial gradient of cells that respond at an ordered sequence of different rates enable time cells and grid cells to develop along a dorsoventral axis in the lateral and medial entorhinal-hippocampal systems, respectively. Populations of time cells can learn how to adaptively time behaviors over time intervals of hundreds of milliseconds, or several seconds. Populations of grid cells can induce learning of hippocampal place cells that can represent the large spaces in which animals navigate. A fourth design explains how and why all neocortical circuits are organized into layers, and how functionally distinct columns develop in these circuits to enable map development. A fifth, and final, design explains the role of Adaptive Resonance Theory top-down matching and attentional circuits in the dynamic stabilization of early development and adult learning.

## Author Contributions

The author confirms being the sole contributor of this work and has approved it for publication.

## Conflict of Interest

The author declares that the research was conducted in the absence of any commercial or financial relationships that could be construed as a potential conflict of interest.

## Footnotes

^1^https://en.wikipedia.org/wiki/Independent_componentanalysis
